# Effects of a virtual voice-based coach delivering problem-solving treatment on emotional distress and brain function: a pilot RCT in depression and anxiety

**DOI:** 10.1038/s41398-023-02462-x

**Published:** 2023-05-12

**Authors:** Thomas Kannampallil, Olusola A. Ajilore, Nan Lv, Joshua M. Smyth, Nancy E. Wittels, Corina R. Ronneberg, Vikas Kumar, Lan Xiao, Susanth Dosala, Amruta Barve, Aifeng Zhang, Kevin C. Tan, Kevin P. Cao, Charmi R. Patel, Ben S. Gerber, Jillian A. Johnson, Emily A. Kringle, Jun Ma

**Affiliations:** 1 Department of Anesthesiology, Washington University School of Medicine, St Louis, MO USA; 2 Institute for Informatics, Washington University School of Medicine, St Louis, MO USA; 3 Department of Psychiatry, University of Illinois at Chicago, Chicago, IL USA; 4 Department of Medicine, University of Illinois at Chicago, Chicago, IL USA; 5 Department of Biobehavioral Health, The Pennsylvania State University, University Park, PA USA; 6 Department of Epidemiology and Population Health, Stanford University, Stanford, USA; 7 Department of Population & Quantitative Health Sciences, University of Massachusetts Medical School, Worcester, MA USA

**Keywords:** Depression, Epigenetics and behaviour

## Abstract

Consumer-based voice assistants have the ability to deliver evidence-based treatment, but their therapeutic potential is largely unknown. In a pilot trial of a virtual voice-based coach, Lumen, delivering problem-solving treatment, adults with mild-to-moderate depression and/or anxiety were randomized to the Lumen intervention (*n* = 42) or waitlist control (*n* = 21). The main outcomes included changes in neural measures of emotional reactivity and cognitive control, and Hospital Anxiety and Depression Scale [HADS] symptom scores over 16 weeks. Participants were 37.8 years (SD = 12.4), 68% women, 25% Black, 24% Latino, and 11% Asian. Activation of the right dlPFC (neural region of interest in cognitive control) decreased in the intervention group but increased in the control group, with an effect size meeting the prespecified threshold for a meaningful effect (Cohen’s *d* = 0.3). Between-group differences in the change in activation of the left dlPFC and bilateral amygdala were observed, but were of smaller magnitude (*d* = 0.2). Change in right dlPFC activation was also meaningfully associated (*r* ≥ 0.4) with changes in self-reported problem-solving ability and avoidance in the intervention. Lumen intervention also led to decreased HADS depression, anxiety, and overall psychological distress scores, with medium effect sizes (Cohen’s *d* = 0.49, 0.51, and 0.55, respectively), compared with the waitlist control group. This pilot trial showed promising effects of a novel digital mental health intervention on cognitive control using neuroimaging and depression and anxiety symptoms, providing foundational evidence for a future confirmatory study.

## Introduction

The prevalence of depression in the United States has increased multiple fold to approximately 32% during the COVID-19 pandemic [1]. Correspondingly, >40 million adults (~19%) have anxiety disorders [2], often with co-morbid depressive symptoms. Efficacious psychotherapies such as problem-solving treatment (PST) exist [3], and in-person PST is a proven intervention for treating both depression and anxiety, which often manifest as comorbid conditions [4–6]. However, access to such therapies is affected by shortages in mental healthcare professionals and high out-of-pocket costs. Digital mental health applications offer viable solutions [7, 8]; consumer-based voice assistants that leverage artificial intelligence to deliver psychotherapy is a nascent and underexplored area with considerable potential for behavioral counseling and to promote emotional well-being [9, 10].

With the integration of voice assistants in mobile devices, their use is pervasive; recent reports have highlighted that nearly a third of search queries rely on voice input [11]. However, their use in healthcare delivery is limited [10], with current research largely focusing on information seeking activities on topics including healthy lifestyle [12, 13], medication names [14], and mental health [15, 16]. Although prototypes of voice-based applications for behavioral assessment and counseling have been developed (e.g., [17, 18]), high-quality clinical research on their therapeutic potential is currently lacking.

Relying on user-centered design principles, and aligned with the treatment fidelity of PST, we developed a virtual voice-based coach, Lumen, that delivers PST for patients with mild-to-moderate depression and/or anxiety [19, 20]. We conducted a pilot randomized clinical trial (RCT) to obtain initial evidence on the effects of PST delivery using Lumen on brain function and clinical outcomes, as is consistent with a theory-driven, mechanism-focused approach to treatment evaluation. The primary objectives were to assess the magnitude of treatment effects on: (a) the activation of a priori neural targets involved in emotional reactivity and cognitive control, the two core theoretical constructs for PST, and (b) depression and anxiety symptoms. We also assessed associations between neural targets and self-reported surveys of emotional reactivity and cognitive control.

## Method

The Institutional Review Board for the University of Illinois Chicago (UIC) approved the study. All participants provided written consent. The study was registered on ClinicalTrials.gov (NCT# 04524104).

### Participants

**Fig. 1:**
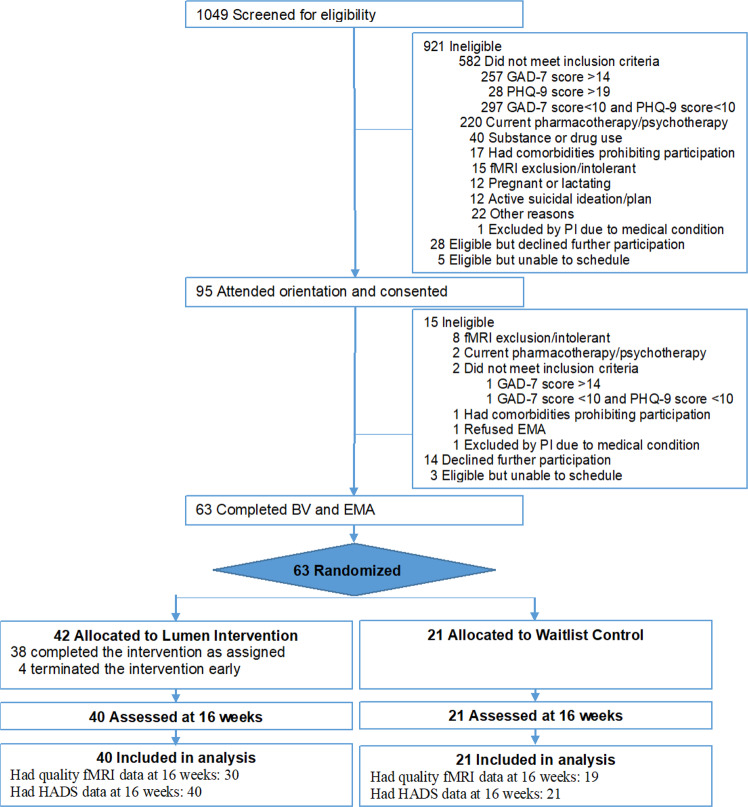
Consort chart. Flowchart regarding the enrollment and randomization of participants.

Enrollment followed a multi-step process (Fig. 1). Participants were recruited between April 5, 2021, and October 7, 2021, from the outpatient care clinics at the University of Illinois Hospital and Health Sciences System (UI Health) and employee email listservs at UIC, a minority-serving institution.

Adults were deemed eligible if they had a Patient Health Questionnaire-9 (PHQ-9) score of 10–19 and/or a Generalized Anxiety Disorder Scale (GAD-7) score of 10–14, without serious medical or psychiatric comorbidities or other exclusions (see Supplementary Material, Section A; also see full protocol in Supplementary Material, Section H). Participants were asked to self-identify their race and ethnicity based on fixed categories to comply with National Institutes of Health’s reporting requirements.

Participants were compensated for this study. As part of this study, participants made two visits for neuroimaging (baseline [visit 1], and at 16 weeks [visit 2]). Upon completion of visit 1, all participants received $50 in compensation. At visit 2, participants in the Lumen intervention arm could choose to receive $100 in compensation or keep the iPad with their access to Lumen deactivated. For those in the waitlist control arm, at visit 2, participants could choose to receive $100 in compensation or choose to attend a Lumen orientation session and receive a Lumen PST-enabled iPad (which they could keep in lieu of the $100 compensation).

### Randomization and masking

Participants were randomly assigned in a 2:1 ratio to receive the Lumen intervention or to be in a waitlist control group using a validated online system [21] based on Pocock’s covariate-adaptive minimization [22]. The 2:1 allocation allowed more participants to receive the Lumen intervention without substantially reducing statistical power [23]. Pocock’s minimization method was used to achieve better-than-chance marginal balance across multiple baseline characteristics: age, sex, race/ethnicity, education, PHQ-9 score, GAD-7 score, and Digital Health Literacy [24]. Investigators, the safety monitor, outcome assessors, and data analysts were blinded to participants’ treatment assignment.

### Lumen

Lumen is a virtual voice-based coach developed on Amazon’s Alexa platform. Lumen delivers an evidence-based PST program [5, 6] consisting of eight sessions (four weekly, followed by four biweekly sessions) for patients with mild-to-moderate depression and/or anxiety. PST is patient-driven, where the coach acts as a guide to identify a problem, set a goal, brainstorm solutions, choose a solution, develop an action plan, and to implement and evaluate the plan [25]. This stepwise approach makes PST appropriate for therapy delivery using a virtual voice-based coach.

Lumen was designed through an iterative user-centered process that involved software developers, interaction designers, psychiatrists, PST experts, and behavioral scientists. Several iterations of the prototype were internally tested; a fully functional prototype underwent feasibility and usability testing with 26 users [19]. The design was driven by two key principles: (a) aligning participants’ voice-based interaction with Lumen similar to the cognitive processes of human communicative interactions [26], and (b) configuring the content of the interactions with the principles and process of evidence-based PST. Towards this end, the Lumen architecture included multiple, interacting components that managed voice-based therapy delivery (a conversation manager), and ascertaining persistence and consistency across the eight therapy sessions (a context manager; see additional information in Supplementary Material sections B, C, and D; Figure S1, Table S1, and Table S2).

For this study, Lumen was integrated within the Alexa app on an iPad provided to all participants. Lumen participants attended an in-person orientation session with a trained health coach where they received their iPad, intervention workbook, and completed a tutorial on how to interact with Lumen. Participants were instructed to begin their PST right away, within 1 week of their orientation session and the health coach helped schedule their 4 weekly and the following 4 biweekly PST sessions. Within 3 days of their first scheduled PST session, the health coach called participants to inquire about any technical issues and helped troubleshoot these issues (if any). Participants received reminder text messages about their upcoming and overdue (if any) PST sessions. Participants with overdue sessions, even after their reminders, were called by the health coach and encouraged to complete their outstanding session(s). Participants also had the opportunity to reach out to the health coach if they faced any issues as part of their study.

For each session, participants instantiated Lumen PST through the Alexa app with a “Launch Lumen Coach” voice instruction and completed their assigned PST sessions. A typical Lumen session lasted ~12 min. Between sessions, participants completed surveys and ecological momentary assessments (EMAs, see Supplementary Materials, Section D, Table S2).

### Waitlist control

Participants in the waitlist control arm received automated text messages to complete surveys and EMAs at intervals similar to the intervention arm. These participants could choose to receive a Lumen-enabled iPad after their end-of-study assessments at 16 weeks.

### Neural target measures

Blinded outcome assessors conducted standardized assessments at baseline and 16 weeks. Task-based functional magnetic resonance imaging (fMRI) data were collected utilizing previously-established standardized fMRI sequences and parameters [27, 28] that inform transdiagnostic phenotypes of neural circuit dysfunction for depression and anxiety. These fMRI methods, including facial expressions task and Go-NoGo tasks, have been standardized in previous work designed for application to precision psychiatry and target engagement studies [29, 30]. A brief description of these tasks are provided below, and additional details can be found in the Supplementary Materials (Section E).

#### Facial expressions task

A standardized set of 3D evoked facial expression stimuli was presented in pseudorandom order, with 5 repeated blocks of 8 stimuli per block for sad, fear, anger, and happy relative to neutral blocks [29]. Participants were instructed to continuously view the faces and were informed beforehand that they would be asked post-scan questions about the faces they were viewing. To assess amygdala activation for the negative affect circuit, our analysis focused on threatening faces only, given our prior research showing threat-related amygdala activation mediating the effect of in-person PST on depression and problem-solving outcomes [31]. Threat stimuli included a combination of fear and anger stimuli relative to neutral blocks. During the conscious viewing condition, each face was presented for 500 ms, with an interstimulus interval of 750 ms. To elicit the negative affect circuit in response to non-conscious threat stimuli, the same fear and anger stimuli were presented in a backward-masking design to prevent awareness. In this non-conscious condition, face stimuli were presented for 10 ms followed immediately by a neutral face mask stimulus for 150 ms, and with a stimulus onset asynchrony of 1250 ms to match that of the conscious condition [32].

#### Go-NoGo task

For the Go-NoGo paradigm, the ‘Go’ and ‘NoGo’ stimuli were presented for 500 ms each with an interstimulus interval of 750 ms. The Go-NoGo paradigm allowed for event-related analysis and is used to assess impulsivity (automatically generated ‘Go’ responses) versus inhibition (‘NoGo’ responses). In the ‘Go’ trials, participants were asked to press a button on GREEN stimuli as quickly as possible (with the word “press” displayed in green); in the ‘NoGo’ trials, participants should withhold button presses on RED stimuli (with the word “press” displayed in red). The probability of ‘NoGo’ stimuli was 0.33. A total of 180 ‘Go’ and 60 ‘NoGo’ stimuli were presented in a pseudorandom order with a constraint to ensure that ‘NoGo’ stimuli were not repeated more than 3 times in a row. Reaction times and number of errors on task were used to evaluate task performance [29].

Informed by previous findings [6, 31] identifying neural targets engaged by in-person PST, the primary target regions of interest (ROIs) were the amygdala (bilaterally) representing a key node in the negative affect circuit, and the dorsal lateral prefrontal cortex (dlPFC) (bilaterally), a key node in the cognitive control circuit. The negative affect circuit was engaged by the viewing of threat faces in the non-conscious viewing condition. The cognitive control circuit was engaged using the Go/No-go task.

Person-level activation of the ROIs for each contrast of interest for each task (e.g., threat versus neutral faces, no-go versus go) was derived in a manner consistent with the methods used in prior studies [27].

### Clinical outcome measures

On the Hospital Anxiety and Depression Scale (HADS) [33, 34], depression and anxiety symptom scores ranged from 0 to 21, with 0–7 indicating normal; 8–10 indicating borderline abnormal (borderline); and 11–21 indicating abnormal (case). HADS total scores were computed as the sum of depression and anxiety scores, indicating overall psychological distress.

### Self-reported measures

Validated self-report surveys of PST theory-based constructs of emotion (affect, worry) and cognition (problem-solving, dysfunctional attitudes) were also completed at baseline and 16 weeks. The Positive and Negative Affect Schedule (PANAS) assessed positive and negative affect [35], with scores ranging from 10 to 50 and higher scores representing higher levels of positive or negative affect. Worry was measured using the Penn State Worry Questionnaire (PSWQ), with a higher total score indicating more worry (range 16–80) [36]. The Social Problem-solving Index-Revised Short Form (SPSI-R:S) assessed total problem-solving ability, with the higher score indicating more productive problem-solving skills, and 5 subscales including problem orientation (positive, negative) and problem-solving styles (rational, impulsive/careless, and avoidant) [37]. Each subscale was scored by summing the respective 5 items (each from 0 to 4), and the total problem-solving ability score ranged from 0 to 20 by averaging the subscale scores. Dysfunctional Attitudes Scale (DAS) measured the presence and intensity of dysfunctional attitudes, with higher scores indicating more dysfunctional attitudes (range 40–280) [38].

### Statistical analysis

The intervention vs. control effects on changes in neural targets and self-reported measures of emotional reactivity and cognitive control from baseline to 16 weeks were assessed using *t* tests. Correlations of changes in neural targets with changes in self-reported measures were estimated using Pearson’s correlation tests.

The intervention vs. control effects on changes in HADS scores from baseline to 16 weeks were tested using ordinary least square regression with adjustment of baseline values of the outcome measure. Each model included all participants with follow-up data on the outcome at 16 weeks, and participants were analyzed based on the group to which they were assigned. Moderation analysis used the same models as above plus the main effect of each potential effect modifier (e.g., sex) and its interaction with the group; the latter, if significant, rejected the null hypothesis of no moderation. Model-adjusted between-treatment mean differences with 95% confidence intervals (CIs) for the overall sample and the subgroups defined by the effect modifiers were reported. Cohen’s d was calculated by the mean difference between the two groups divided by the pooled standard deviation.

Given that this study was a pilot RCT, the primary purpose was to establish a reliable signal regarding the impact of Lumen on neural targets and clinical outcomes that would be promising enough to warrant further research. Towards this end, we used Cohen’s *d* ≥ 0.3 to define the meaningful mean difference between the intervention and control groups in neural target and symptom changes from baseline to 16 weeks. Moreover, our approach to data reporting and interpretation regarding the intervention effects on neural targets and symptom outcomes was focused on the magnitude and precision (95% CI) of the effect estimates, and not on *p*-values [5]. Similarly, we were not focused on smaller correlations (Pearson’s *r* < 0.4) between the neural targets and self-reported measures as it would have limited clinical relevance.

All analyses were conducted using SAS, version 9.4 (SAS Institute Inc., Cary, North Carolina).

### Sample size calculation

The sample size of this pilot RCT was calculated using a confidence interval approach. To obtain a precision interval with a standardized half-width of 0.50 (akin to a medium effect) with 90% assurance, we had planned a sample size of 60 (*n*_*Treatment*_ = 40, *n*_*Control*_ = 20), assuming ≥85% retention at 16 weeks. A precision interval approach was used where we defined that, compared with the waitlist control group, the intervention group will demonstrate a meaningful improvement in outcomes (in both neural targets and symptoms) if the standardized between-group mean difference was at least Cohen’s d = 0.3 in favor of intervention. At this effect size, the upper limit of the precision interval overlaps with *d* = 0.8 (large effect) given a standardized half-width of 0.5 with 90% assurance that the interval contains the true mean difference based on power analysis. For the correlation of change in neural targets with change in self-reported measures, a sample size of 51 (i.e., 60 × 85%) would be sufficient to detect a coefficient of r = 0.4 with 80% power and 2-sided α = 0.05.

## Results

### Sample characteristics and retention

Of 1049 individuals who completed screening, 936 were ineligible and 50 declined or were unable to participate (Fig. 1). Randomized participants included 42 in the intervention and 21 in the waitlist control. Participants had a mean age of 37.8 years (SD = 12.4), 68% were women, 25% were Black, 24% were Latino, 59% had a high school or college (1 to 4+ years) education, and 51% had an annual income less than $55,000 (Table 1). On average, participants had moderate depression (mean PHQ-9 Score=12.8 [SD = 3.1]) and anxiety (mean GAD-7 Score=9.7 [SD = 2.7]), and borderline abnormal HADS depression scores (mean HADS depression = 7.3 [SD = 3.0]), and abnormal HADS anxiety scores (mean HADS anxiety Score=10.6 [SD = 3.3]). Participants had a total HADS score of 17.9 [SD = 5.2]. Based on PHQ-9 and GAD-7 categories of symptom severity, a majority of the 63 participants had moderate to moderately severe depression or moderate anxiety, and 29 had both. All 63 participants had complete baseline data, and 61 (97%) were assessed at 16 weeks. Of the 42 Lumen participants, 38 (90.5%) completed at least 4 PST sessions, and 34 (81.0%) completed all 8 PST sessions.

**Table 1:** Eligibility screening and sociodemographic measures^a^; the prognostic factors^b^ for randomization included age, sex, race/ethnicity, educational level, PHQ-9 score, and GAD-7 score.

Characteristic	All participants (*n* = 63)	Intervention (*n* = 42)	Control (*n* = 21)
Age, years^b^	37.8 ± 12.4	38.9 ± 12.9	35.6 ± 11.5
Female, %^b^	43 (68.3)	28 (66.7)	15 (71.4)
Race/Ethnicity (%)^b^			
Non-Hispanic White	22 (34.9)	15 (35.7)	7 (33.3)
African American	16 (25.4)	8 (19.1)	8 (38.1)
Asian/Pacific Islander	7 (11.1)	5 (11.9)	2 (9.5)
Hispanic	15 (23.8)	12 (28.6)	3 (14.3)
Other (e.g., declined to state, multirace)	3 (4.8)	2 (4.7)	1 (4.8)
Education (%)^b^			
High school/GED or less	2 (3.2)	1 (2.4)	1 (4.8)
College - 1 year to 3 years	14 (22.2)	10 (23.8)	4 (19.1)
College - 4 years or more	21 (33.3)	17 (40.5)	4 (19.1)
Post college	26 (41.3)	14 (33.3)	12 (57.1)
Income (%)			
< $35,000	17 (27.0)	9 (21.4)	8 (38.1)
$35,000–<$55,000	15 (23.8)	10 (23.8)	5 (23.8)
$55,000–<$75,000	8 (12.7)	6 (14.3)	2 (9.5)
>=$75,000	23 (36.5)	17 (40.5)	6 (28.6)
PHQ-9 score^b^	12.8 ± 3.1	12.7 ± 3.0	13 ± 3.3
Mild depression 5–9 (%)	7 (11.1)	4 (9.5)	3 (14.3)
Moderate depression 10–14 (%)	38 (60.3)	27 (64.3)	11 (52.4)
Moderately severe depression 15–19 (%)	18 (28.6)	11 (26.2)	7 (33.3)
GAD-7 score^b^	9.7 ± 2.7	9.8 ± 2.5	9.4 ± 3.0
Minimal anxiety 0–4 (%)	1 (1.6)	0 (0.0)	1 (4.8)
Mild anxiety 5–9 (%)	26 (41.3)	17 (40.5)	9 (42.9)
moderate anxiety 10–14 (%)	36 (57.1)	25 (59.5)	11 (52.4)
HADS Depression score	7.3 ± 3.0	7.6 ± 2.9	6.7 ± 3.2
HADS Anxiety score	10.6 ± 3.3	11.0 ± 2.7	9.9 ± 4.1
HADS Total score	17.9 ± 5.2	18.6 ± 4.3	16.6 ± 6.6
Digital Health Literacy (%)^b^			
Low 1–1.999	0 (0.0)	0 (0.0)	0 (0.0)
Medium 2–2.999	11 (17.5)	7 (16.7)	4 (19.0)
High 3–4	52 (82.5)	35 (83.3)	17 (81.0)

*GAD-7* Generalized Anxiety Disorder-7, *GED* general educational development, *HADS* Hospital Anxiety and Depression Scale, *PHQ-9* Patient Health Questionnaire-9.

^a^Values are mean ± SD unless noted otherwise.

^b^Prognostic factors for randomization: age, sex, race/ethnicity, education, digital health literacy, PHQ-9, and GAD-7.

### Intervention effect on activation of neural targets

Between-group mean differences in changes in the primary ROIs (i.e., activation decrease in the control group > intervention in L. amygdala, activation decrease in intervention group > control group) engaged in the non-conscious threat stimuli (i.e., emotional reactivity) from baseline to 16 weeks did not meet the Cohen’s d = 0.3 threshold. There was, however, a meaningful change in a primary cognitive control target: the activation of both the right and left dlPFC decreased in the intervention from baseline to 16 weeks compared with the control, while the between-group mean difference in activation of the right dlPFC (−0.20 [95%CI: −0.61, 0.22]) met Cohen’s d = 0.3 (Table 2).

**Table 2:** Treatment effects on primary neural target regions of interest and self-reported measures^a,b^.

Circuit	Target Measure	Baseline		Change at 16 weeks from Baseline			Cohen’s d
		Intervention	Control	Intervention	Control	Mean difference (95% CI)	
Neural target regions of interest							
Negative Affect	Amygdala L	0.06 ± 0.19	0.06 ± 0.21	−0.07 ± 0.39	−0.13 ± 0.29	0.05(−0.16, 0.27)	0.2
	Amygdala R	0.05 ± 0.19	0.07 ± 0.31	−0.14 ± 0.44	−0.07 ± 0.40	−0.07(−0.33, 0.19)	0.2
Cognitive Control	dlPFC L	0.23 ± 0.41	0.22 ± 0.38	−0.02 ± 0.49	0.09 ± 0.43	−0.11(−0.39, 0.17)	0.2
	dlPFC R	0.58 ± 0.60	0.52 ± 0.59	−0.10 ± 0.64	0.09 ± 0.78	−0.2(−0.61, 0.22)	**0.3**
Self-reported measures							
Negative Affect	Positive Affect Score^c^	25.21 ± 6.26	27.43 ± 6.74	4.03 ± 7.79	2.43 ± 7.89	1.6(−2.62, 5.82)	0.2
	Negative Affect Score^d^	27.43 ± 6.11	25.57 ± 8.41	−1.60 ± 9.67	−0.90 ± 7.58	−0.7(−5.56, 4.17)	0.1
	Penn State Worry Questionnaire^e^	60.69 ± 11.22	59.14 ± 11.46	−3.90 ± 9.99	−3.95 ± 11.01	0.05(−5.53, 5.63)	0.0
Cognitive Control	SPSI-R:S raw score^f^	11.27 ± 2.99	12.48 ± 2.74	0.83 ± 2.92	0.42 ± 2.19	0.41(−1.04, 1.86)	0.2
	PPO raw score^f^	10.52 ± 4.39	11.05 ± 4.48	0.60 ± 4.39	1.62 ± 3.51	−1.02(−3.24, 1.2)	0.2
	NPO raw score^f^	9.69 ± 3.53	8.00 ± 4.80	−1.03 ± 4.59	−0.62 ± 3.67	−0.41(−2.72, 1.91)	0.1
	RPS raw score^f^	9.71 ± 4.22	10.86 ± 3.82	1.95 ± 4.61	0.52 ± 5.57	1.43(−1.25, 4.1)	**0.3**
	ICS raw score^f^	6.24 ± 4.12	5.29 ± 3.89	−0.43 ± 4.06	1.05 ± 3.56	−1.47(−3.57, 0.63)	**0.4**
	AS raw score^f^	7.95 ± 5.52	6.24 ± 4.35	−0.15 ± 4.09	−0.38 ± 4.33	0.23(−2.02, 2.48)	0.1
	Dysfunctional Attitudes Scale^g^	140.0 ± 37.54	128.4 ± 38.36	−12.8 ± 30.39	−6.62 ± 20.49	−6.16(−20.95, 8.64)	0.2

*SPSI-R:S* Social Problem-solving Index-Revised Short Form, *PPO* positive problem orientation, *NPO* negative problem orientation, *RPS* rational problem-solving style, *ICS* impulsive/careless problem-solving style, *AS* avoidant problem-solving style.

^a^*t* tests.

^b^Values are mean ± SD unless otherwise noted.

^c^Scores range from 10 to 50, with higher scores representing higher levels of positive affect.

^d^Scores range from 10 to 50, with lower scores representing lower levels of negative affect.

^e^The total score of the scale ranges from 16 to 80, with higher score indicating more worry.

^f^SPSI-R:S score = (PPO raw score/5)+(20- NPO raw score)/5 + (RPS raw score/5)+(20- ICS raw score)/5 + (20- AS raw score)/5; the higher the score the more productive overall problem-solving orientation and skills. Subscales (PPO, NPO, RPS, ICS, and AS) are raw scores without reversal.

^g^Scores range from 40 – 280, with higher score indicating more dysfunctional the subject’s attitudes.

### Intervention effect on clinical outcomes

At 16 weeks, intervention participants had greater improvements in their HADS depression, anxiety and total scores compared with control participants, with a medium effect size. Model-adjusted between-group mean difference was −1.33 (95%CI: −3.26, 0.60; Cohen’s d = 0.49) for the HADS depression score, −1.58 (95%CI: −3.82, 0.66; Cohen’s d = 0.51) for the HADS anxiety score, and −2.89 (95%CI: −6.76, 0.99; Cohen’s d = 0.55) for the HADS total score (Table 3).

**Table 3:** Treatment effects on depression and anxiety symptoms.

	Unadjusted mean ± SD			Model-based mean difference (95%CI)*	Cohen’s d
Symptom	Intervention	Control	P value		
HADS_Depresson^a^					
baseline	7.62 ± 2.89	6.67 ± 3.15	0.24		
change at 16 weeks	−1.85 ± 4.00	0.10 ± 3.95	0.075	−1.33(−3.26, 0.6)	0.49
HADS_Anxiety^a^					
baseline	10.98 ± 2.72	9.90 ± 4.15	0.29		
change at 16 weeks	−2.25 ± 4.48	0.14 ± 5.11	0.064	−1.58(−3.82, 0.66)	0.51
HADS_Total^b^					
baseline	18.60 ± 4.30	16.57 ± 6.60	0.21		
change at 16 weeks	−4.10 ± 7.53	0.24 ± 8.49	0.045	−2.89(−6.76, 0.99)	0.55

^*^Regression model adjusted for baseline value of the interest.

^a^Scores range from 0 to 21, with 0–7 = Normal; 8–10 = Borderline abnormal (borderline case); 11–21 = Abnormal (case).

^b^Scores range from 0 to 42.

**Fig. 2:**
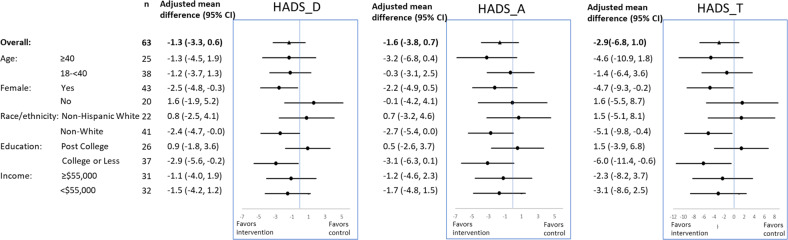
Treatment effect on symptoms. Treatment effects on HADS depression score (HADS_D), HADS anxiety score (HADS_A) and total HADS score (HADS_T), overall and by sub-group.

The treatment effect on the HADS depression score was significantly moderated by sex (*p* = 0.048), education (*p* = 0.048), and digital health literacy score (*p* = 0.03). The Lumen group had consistently greater improvements than the control group in HADS depression, anxiety, and total scores than control participants among women (between-group mean difference [95% CI]: −2.5 [−4.8, −0.3], −2.2 [−4.9, 0.5], −4.7 [−9.3, −0.2], respectively), non-White (−2.4 [−4.7, −0.0], −2.7 [−5.4, 0.0], −5.1 [−9.8, −0.4], respectively), and those with college or less education (−2.9 [−5.6, −0.2], −3.1 [−6.3, 0.1], −6.0 [−11.4, −0.6], respectively) at 16 weeks (Fig. 2).

In addition, participants with lower digital health literacy scores had a greater mean decrease in their HADS depression score in the Lumen vs. control group (Supplementary Material, Section F, Figure S2).

### Intervention effect on self-reports

Each of the self-reports associated with emotional reactivity (positive affect score, negative affect score, Penn State worry questionnaire) showed improvement in the intervention and control groups from baseline to 16 weeks, but did not show meaningful between-group differences (all Cohen’s d < 0.3). Similarly, all self-reports associated with cognitive control showed improvement over time, with the rational problem-solving (1.43 [95%CI: −1.25, 4.1], Cohen’s d = 0.3) and impulsive/careless raw scores (−1.47, [95%CI: −3.57, 0.63], Cohen’s d = 0.4) having meaningful differences in improvement in the intervention group compared with the control (Table 2).

### Association of neural targets and self-reports

In the intervention group, an increase in the activation of the right dlPFC was positively correlated with an increased self-reported SPSI-R score indicating improved problem-solving ability (*r* = 0.4, *p* = 0.02), and negatively associated with the self-reported avoidant score indicating reduced avoidance (*r* = −0.5, *p* = 0.01), from baseline to 16 weeks. In the waitlist control group, the right dlPFC was negatively correlated with dysfunctional attitudes score (*r* = −0.5, *p* = 0.04). Moreover, associations were in the opposite direction in the control group for several of the other considered scales, although they were not statistically significant: negative correlation with the self-reported SPSI-R score (*r* = −0.5, *p* = 0.050), and positive correlation with the self-reported avoidant score (*r* = 0.3, *p* = 0.20) (see Supplementary Materials, Section G, Tables S3 and S4; Figure S3).

## Discussion

A virtual voice-based coach intervention showed meaningful changes in a subset of select neural targets, with a decrease in the activation of the primary neural target related to cognitive control—the right dlPFC—in the intervention group compared with the control. Related self-reported rational problem-solving and impulsive/carelessness scores also showed meaningful improvements with Lumen. The change in the right dlPFC activation was also correlated positively with self-reported problem-solving ability scores and negatively with the avoidance scores in the Lumen group. Moreover, participants in the Lumen intervention group showed improvements in both depression and anxiety symptoms as well as total psychological distress at 16 weeks, compared with the waitlist control group. The between-group differences in the HADS-D and HADS-A scores were consistently greater than clinically important differences defined as 1.5–1.7 in prior studies [39, 40]. These treatment effects were moderated by participant sex, race/ethnicity, and educational status. These findings offer a consistent signal and support the pragmatic viability of Lumen as a promising digital intervention to address mild-to-moderate depression and/or anxiety.

To the best of our knowledge, this is the first clinical trial of a virtual voice-based coach for behavioral therapy, coupled with neuroimaging, that was delivered using a consumer-based voice platform (i.e., Amazon’s Alexa). The demonstration of neural target engagement for cognitive control and improved clinical outcomes is promising and offers considerable opportunity for delivering PST. Moreover, more than 80% of the Lumen participants completed all 8 PST sessions, suggesting high feasibility and acceptability among a highly diverse group of participants.

The neural mechanistic findings supplement the validated self-report clinical outcomes, and may help elucidate the theory-based therapeutic underpinnings of this novel form of PST delivery. Meaningful decrease in the activation of right dlPFC in the Lumen group is consistent with other studies suggesting that right hemisphere hyperactivation is associated with depressive disorders [41]. Furthermore, the right dlPFC is frequently used as a target for low-frequency inhibitory repetitive transcranial magnetic stimulation, suggesting that a reduction of activity in this region has an antidepressant effect [42]. The association of right dlPFC activation with increased problem-solving skills and decreased avoidant scores in the intervention group may represent efficient processing by a key node involved in the cognitive control circuit. This finding, in combination with our prior work from the ENGAGE-2 study [6], suggests that the malleability of dlPFC activity may serve as a prognostic biomarker for identifying patients who would likely respond to this type of intervention.

The presence of moderators in our models suggests that women, minorities, those college or less educated or those with lower digital health literacy may likely benefit more from using Lumen. Among marginalized groups with lower resources and limited access to mental health services, Lumen offers a potential resource for easy and on-demand access. This is especially the case, given the significant proliferation of mobile phones with voice applications. A recent qualitative study [43] found similar results, showing the potential benefits and opportunities for using virtual technology for health management among Black men, further emphasizing the role of digital tools among minority populations.

There is considerable published literature and on-going research on the use of text-based applications (“chatbots”) for mental health support [44, 45]; however, a recent meta-analysis showed that text-based chatbots had mixed results [46]. In this context, our study using a virtual voice-based coach offers a novel mental health therapy delivery mechanism. The absence of full-fledged RCTs of voice-based applications is likely due to the challenges high-quality natural language understanding on these platforms. However, it must be stated that these platforms have had greater success in directed and specific conversations utilizing the extensive modern machine learning and natural language processing algorithms. As such, Lumen was designed to string together multiple short, directed conversations (e.g., “what is your goal?”) that reflect the therapeutic approach underlying the delivery of PST [19].

This study has several limitations. First, this was a pilot RCT with a small study sample, among those with mild-to-moderate depression and/or anxiety, increasing the probability of false discovery and failure to detect uncommon problems or adverse events. Nonetheless, this study provides the foundational evidence for a planned confirmatory study (NCT05603923). Second, task-based neuroimaging studies have had varying within-subjects reliability, which may have reduced the capacity to detect changes in certain neural targets. Future studies will also complement data-driven approaches such as whole-brain network analysis. Third, the comparison group did not receive any treatment, and it remains unclear how Lumen differs from PST delivered by human coaches. Fourth, we considered several moderator variables in our analysis; given the small sample size the findings should be considered preliminary. Finally, although education and digital literacy were moderating variables, the overall sample was well-educated and digitally literate.

In summary, this pilot RCT provides preliminary evidence that a virtual voice-based coach may represent an alternative option of PST delivery for managing mild-to-moderate depression and anxiety. This innovative approach may reduce barriers to mental health care access, particularly for vulnerable populations.

## Supplementary information

Supplemental Material

## Data Availability

Data used in the preparation of this manuscript will be submitted to the National Institute of Mental Health (NIMH) Data Archive (NDA). NDA is a collaborative informatics system created by the National Institutes of Health to provide a national resource to support and accelerate research in mental health. Those wishing to use this data can contact the corresponding author for the dataset identifier and make a request to the NIMH (visit https://nda.nih.gov/). This manuscript reflects the views of the authors and may not reflect the opinions or views of the NIH.
