# Correction: Effects of a virtual voice-based coach delivering problem-solving treatment on emotional distress and brain function: a pilot RCT in depression and anxiety

**DOI:** 10.1038/s41398-023-02544-w

**Published:** 2023-07-04

**Authors:** Thomas Kannampallil, Olusola A. Ajilore, Nan Lv, Joshua M. Smyth, Nancy E. Wittels, Corina R. Ronneberg, Vikas Kumar, Lan Xiao, Susanth Dosala, Amruta Barve, Aifeng Zhang, Kevin C. Tan, Kevin P. Cao, Charmi R. Patel, Ben S. Gerber, Jillian A. Johnson, Emily A. Kringle, Jun Ma

**Affiliations:** 1 Department of Anesthesiology, Washington University School of Medicine, St Louis, MO USA; 2 Institute for Informatics, Washington University School of Medicine, St Louis, MO USA; 3 Department of Psychiatry, University of Illinois at Chicago, Chicago, IL USA; 4 Department of Medicine, University of Illinois at Chicago, Chicago, IL USA; 5 Department of Biobehavioral Health, The Pennsylvania State University, University Park, PA USA; 6 Department of Epidemiology and Population Health, Stanford University, Stanford, USA; 7 Department of Population & Quantitative Health Sciences, University of Massachusetts Medical School, Worcester, MA USA

**Keywords:** Depression, Epigenetics and behaviour

Correction to: *Translational Psychiatry* 10.1038/s41398-023-02462-x, published online 12 May 2023

In this article the author name Kevin P. Cao was incorrectly written as Kevin K. Cao.

The original article has been corrected.

