# Supplementary material for: Effects of a virtual voice-based coach delivering problem-solving treatment on emotional distress and brain function: a pilot RCT in depression and anxiety
Source: Transl Psychiatry. 2023 May 12;13:166. doi: 10.1038/s41398-023-02462-x (PMC10175049; doi:10.1038/s41398-023-02462-x)
Supplement: Supplementary file 1 — Supplemental Material [file 41398_2023_2462_MOESM1_ESM.docx]

Supplementary Material

# Section A: Inclusion and Exclusion Criteria

# *Inclusion Criteria*

# Age: ≥ 18 years

# Emotional distress defined by elevated depressive (PHQ-9 scores 10-19) and/or anxious symptoms (GAD-7 scores 10-14)

# Willing and able to provide informed eConsent and HIPAA authorization

# *Exclusion Criteria*

# Unable to speak, read, or understand English for informed consent

# Current pharmacotherapy or psychotherapy (individual or professionally led group therapy) for depression or anxiety (note: participants are not withdrawn post-randomization if they begin pharmacotherapy drugs or start psychotherapy during the study.)

# Suicidal ideation per PHQ-9 with active plan

# Bipolar or psychotic disorder, or current psychiatric treatment

# Weight ≥325 pounds due to brain scanner constraints, MRI contraindications, traumatic brain injuries, and tumor or any other known structural abnormality in the brain

# Severe medical condition (e.g., myocardial infarction or stroke or new cancer diagnosis in the past 6 months, end-stage organ failure, terminal illness) or residence in a long-term care facility

# Diagnosis of cancer (other than non-melanoma skin cancer) that is/was active or treated with radiation or chemotherapy within the past year

# Active alcohol or substance use disorder (including prescription drugs) based on the CAGE Questionnaire Adapted to Include Drugs (CAGE-AID)

# Cognitive impairment based on the Callahan 6-item screener

# Current or planned pregnancy or lactating (<6 months postpartum)

# Participation in other investigational treatment studies that would significantly affect participation in this study, raise safety concerns, and/or confound outcomes (participant may be asked to provide the informed consent of the other study for final decision on exclusion by a study psychiatrist)

# Family/household member of an already enrolled participant or of a study team member

# Plan to move out of the Chicago area during the study period

# Does not have reliable Wi-Fi Internet at home

# Unwillingness to user personal mobile device to receive study text messages

# Investigator discretion for clinical safety or protocol adherence reasons

# Section B: Lumen Architecture

Lumen is a virtual voice-based coach that delivers Problem Solving Treatment (PST) to counsel participants with depression and/or anxiety using the 7-step problem solving process and the SSTA (stop, slow down, think and act) method of coping. Lumen conducts interactive conversations providing appropriate responses based on participant input. In order to deliver seamless interactions, we developed a robust and parsimonious architecture that combines PST, integrated components to provide context for Lumen conversations, data storage for the interactions, and a software infrastructure for providing security and privacy for these interactions.

Lumen architecture (see **Figure S1**) was developed with input and consultation from researchers (in computer science, medicine, health service researchers, and psychiatry), software developers, and human computer interaction experts. The architecture was developed on Amazon’s Alexa platform, with further integration with a secure REDCap system for ecological momentary assessments (EMAs) and surveys. Lumen’s software architecture is comprised of two components: a conversation manager module and a context manager module.

**Figure S1. Lumen software architecture that includes the conversation manager and context manager modules.**

The **conversation manager** is the voice-interactive component of Lumen and is responsible for delivering PST content. This PST content is aligned with the theoretical constructs and associated treatment guidelines. Towards this end, the conversation manager includes a PST engine that incorporates PST-related and conversational structures. The PST engine is a flexible set up that allows for the adaptation of PST content for other health-related problems (e.g., translating the therapy for weight management or smoking cessation) in the future.

Within the Lumen application, the PST engine tracks the progress of a PST session. For example, in a session, once the participant defines a specific problem to work on (step 1), Lumen will proceed to guide the participant in establishing a realistic goal for solving the problem (step 2). Then, once the participant has defined a set of potential solutions that can meet the goal (step 3), Lumen will guide the participant on evaluation of the pros and cons of each solution (step 4). After the participant chooses his/her preferred solution (step 5), Lumen will guide the participant to develop an action plan (step 6), which Lumen will prompt the participant to implement and evaluate the outcome post the session (step 7). This stepwise structure is the same in all PST sessions as is the case in current practice, and the participant identifies a problem to work on in each session (except session 1, which is an introductory overview session situating the PST process and building an initial problems list which participants can choose to work on in subsequent sessions, or they can opt to work on a new problem). Importantly, each of these steps is based on established PST theory and practice.

In addition, the conversation manager also manages the “state” of the conversation—including the flow of interactive conversation (e.g., the flow of the above-mentioned steps), and adaptive responses based on user input. The conversational state is adaptively managed based on user input. Towards this end, user “intents” or statements are parsed and mapped into pre-defined categories, which are then mapped to the appropriate state in the PST interaction to deliver relevant content, aligned with the user input. The conversation manager also provides dynamic support for functions such as resume, which allows participants to re-start a previously incomplete PST session. Such stop-restart functions provide flexibility in conversational interactions, affording perceptions of realistic conversational interactions.

The conversation manager also interacts with a **context manager** module that provides situated and contextual information regarding Lumen sessions. The context manager primarily controls three aspects: persistence of therapy content across sessions, scheduling/re-scheduling of sessions, and integrating external content (e.g., surveys, EMAs) into the Lumen sessions. The context manager dynamically tracks user identified problems (see step 1 above), and goals that were previously developed and asks participants to self-evaluate adherence to their previously developed action plans in ensuing sessions. Such dynamic follow-up increases the persistence and continuity of therapy across sessions, developing trust and confidence in the virtual coach. Similarly, the context manager tracks session progress (e.g., session 5), and helps participants schedule and re-schedule sessions. With its integration with an external scheduling database (in REDCap), follow-up emails and messages are tracked to ensure that the therapy sessions are synchronized with participant needs. Finally, the context manager also interacts with the REDCap database to incorporate survey responses (e.g., completion of the PHQ-9 and GAD-7 surveys before each session) into the Lumen session. For example, prior to the start of each Lumen session, participants are asked to complete the PHQ-9 and GAD-7 surveys; if incomplete (or partially complete), Lumen prompts the user to complete the surveys then re-start the session.

All sessions and communicative interactions are stored in a secure AWS-based database for analysis. In order to prevent accidental recording, and for pragmatic implementation in a clinical trial, we currently have implemented the entire Lumen infrastructure in a “locked down” mode in an 8^th^ generation Apple iPad. Participants can access the Lumen application skill within the Alexa application, preloaded on their study iPad, by stating “Alexa, Open Lumen….” A summary of the Lumen components is provided in Table S1.

**Table S1. Lumen components, and their associated functions.**

| **Lumen Component** | **Functions** |
| --- | --- |
| Conversational manager | Managing conversations based on user intent, aligning with PST constructs, additional functions to manage conversations (e.g., repeat, resume), tracking progress within a session |
| Context manager | Tracking user problems and goals (from previous PST sessions), prompting participants to self-evaluate action plan adherence, integrating user responses from PHQ-9 and GAD-7 surveys within sessions, scheduling/re-scheduling sessions, |
| Lumen REDCap database | Ecological daily assessments, patient surveys, patient calendaring and reminders |
| Storage/Data Management (AWS & REDCap) | Comprehensive storage of user responses to surveys, user interactions with the Lumen, synchronization across devices, data security and privacy |

# Section C: Interacting with Lumen

Lumen architecture that is presented in Section A, is realized through user interactions with the Lumen skill embedded within the Alexa application (currently delivered on an iPad device) and user completion of surveys and EMAs with hyperlinks delivered via emails and text messages (on the participant mobile phones).

Participant interaction with the Lumen PST coach involves two primary components: (a) sessions with the coach, and (b) completion of surveys, EMAs, administrative management (e.g., session scheduling, re-scheduling, monitoring progress on their personal dashboard).

Participants access their scheduled Lumen session via the Alexa application on their assigned iPad. Sessions are instantiated by the participant by saying “Alexa, Open Lumen.” Conversations then continue based on the progress that participants have made (e.g., number of sessions completed), current problem(s) being addressed and implementation of previously created goals and action plans. As previously described, these conversations are aligned with PST’s treatment protocol and guidelines and with conversational structures and flow that reinforce the patient-centered approach of PST. As with regular conversations, participants can exit, resume or ask the Lumen coach to repeat parts of the conversations that they are unable to follow. At the end of each session, participants schedule their next session with Lumen.

Lumen’s PST sessions are delivered on a dedicated iPad, which is encrypted and functions in a “lockdown” mode with no additional functions or local storage. The purpose of using such an approach is for trialing it in a controlled environment without compromising on data safety and privacy of the user. Additionally, such an infrastructure and set-up provide several advantages. First, it creates the perception of a “device as a therapist” mode, assigning a specific role-based purpose (i.e., Lumen as a health coach, that is delivered only on the specific device). Second, embedding the Lumen skill within the Alexa application reduces the potential for accidental recording (as is common with smart speaker devices such as Echo or Google Assistant). Finally, the encrypted mode with no local data storage allows for data privacy protection and remote management, in case of a lost or misplaced device.

Between sessions, participants will also complete surveys and EMAs to track their progress. These administrative tasks and surveys, which provide situated context for Lumen interaction and monitoring of progress will be delivered on the participant’s personal devices. The surveys and EMAs are sent by text message with embedded links. Participants complete these on any browser associated with their mobile phone. Additional messages will include reminders about sessions, ability to schedule/re-schedule planned sessions, and other session related information.

All of the survey, EMA and scheduling tasks are instantiated through a dedicated REDCap project. Based on an initial session date, a program calendar according to PST guidelines (4 weekly, then 4 biweekly sessions) is set up and used for all session reminders. Future session appointments are confirmed or adjusted per user preference during Lumen sessions and are automatically updated in REDCap to assure that PHQ-9 and GAD-7 surveys are delivered at appropriate times.

# Section D: Components of PST Implemented in Lumen

To align with the treatment fidelity of the evidence-based PST, components of PST were implemented within the conversation manager and context manager of Lumen. The timing of the delivery of these components were aligned with the evidence-based PST. Each of the PST components, the timing of the delivery (i.e., session), and the aspects of the component managed by the conversation manager and context manager are provided in Table S2.

**Table S2. Components of PST that were implemented in various sessions within the conversation and context managers of Lumen, respectively.**

| **PST Fidelity Components** | **Session** | **Lumen Conversation Manager** | **Lumen Context Management (survey, EMA, participant workbook)** |
| --- | --- | --- | --- |
| **Introduction to Lumen and Building Rapport** | **Session 1** | Lumen introduces and tells participants about its role and purpose Personalized information | • Utilizes participant specific information stored in a profile |
| **Introduction to the Program and PST Overview** | **Session 1** | Natural conversation and verbal description of Lumen, background on PST and how it works |  |
| **Administer and Verify Completion of PHQ-9 and GAD-7** | **All Sessions** | Lumen checks and validates if surveys are completed within past 3 days, and, if not completed, provides directions on how to complete them | • REDCap links to complete PHQ-9/GAD-7 surveys externally sent via SMS. • Survey reminders sent before each session  • If session with Lumen begins and the PHQ-9/GAD-7 surveys are not complete, participant is asked to exit the session, complete surveys, then resume session • If PHQ-9/GAD-7 surveys are not completed within 3 days of completing Lumen session, participants sent new link and asked to re-take surveys. • REDCap links to complete brief (2-3 minute) EMAs that assess mood are sent for 7 days in a row, every other week. EMAs are open from 7PM to midnight on the day they are sent. If an EMA is not filled out by 9:30PM on the day it is sent, participants will receive one reminder to complete it. |
| **Knowledge Check** | **Session 1** | Multiple Choice questions asked by Lumen to evaluate participant understanding of key concepts before problem solving begins (e.g., link between unresolved problems and emotional distress, number of skills to be learned in the program, following a person-centered approach, rationale for utilizing PHQ-9/GAD-7, behavioral activation and planning varied activities) |  |
| **Problem List Generation** | **Session 1** | Lumen prompts participants to list problems they would like to work on in subsequent sessions | • Participants fill out the "Problem List" page in their workbook. |
| **7-Step PST Process** | **Sessions 2-8** | Lumen verbally walks participants through the steps of problem solving (Problem Identification, Goal Selection, Generating Possible Solutions, Solution Evaluation, Solution Choice, Action Plan Development). Lumen confirms (by repeating) participant answers and guides them to self-evaluate their responses at each step. Participants are asked if they need additional time as they complete each step.  Lumen will recall selected problems and goals and will ask participant about their action plan implementation at the next session as part of the progress review. | • Participants fill out "Problem Solving Worksheet" in their workbook to keep track of their listed problems. |
| **Progress Review** | **Sessions 3-8** | Prior to starting a new problem-solving session, Lumen asks participants to evaluate their action plan implementation from the immediate previous session  Lumen reminds participants of problems and goals they’ve chosen in the previous session. Further follow up is also conducted regarding how the participant was able to implement and adhere to the action plan, with Lumen prompting the participant to self-evaluate their action plan adherence. Additional questions regarding whether planned activities (Behavioral Activation) were carried out are also asked. | • Fill out "Progress Review" section of the workbook |
| **Behavioral Activation** | **Sessions 2-8** | Lumen explains 3 activities (social, physical, pleasant), their importance, along with examples. Participants are guided through planning 1 of each type of activities to partake in by the next session.  Lumen confirms participant activity choice and will ask participants whether they were able to complete their scheduled activities, at the next session, as part of the progress review | • Fill out "Behavioral Activation Worksheet" section of the workbook |
| **Scheduling, Rescheduling, Reminders** | **Between each session** | Participants are sent text message reminders about upcoming Lumen sessions (2/day) and asked to complete PHQ-9 and GAD-7 surveys before starting their upcoming Lumen session.  The timing of the sessions are based on session 1 (Lumen asks participant to write these down on the "Lumen Sessions" page in their workbook). Participants are encouraged to follow the pre-arranged session schedule; in case of emergency, participants have the ability to reschedule their Lumen session to a day before or after their pre-arranged session schedule. |  |

**Note that the contents in this supplementary material Sections A through C, have been previously described in the primary Lumen design paper.(1)

# Section E: Neural Targets

Imaging Sequences

BOLD contrast functional images were acquired with echo-planar T2*-weighted imaging using a GE MR750 3T scanner (GE Healthcare, Milwaukee, Wisconsin) with a NOVA 32-channel head coil. Head motion was restricted with foam pads.

Each whole brain volume consisted of 45 interleaved 3mm thick axial/oblique slices (74 x 74 matrix; TR=2000ms; TE=27.5ms; voxel size=3x3x3mm; FOV=222mm; flip angle=77°). One hundred fifty-four volumes were acquired over 5 minutes and 8 seconds for both tasks. A high-resolution T1-weighted structural scan was acquired using GE’s BRAVO sequence at the end of the imaging session for use in normalization of the fMRI data into standard space with the following parameters: TR=0.008, TE=0.003; voxel size=1x1x1mm; number of slices=176; FOV=256x256; flip angle=11^o^.

Image Pre-processing

Pre-processing and data analysis were performed using Statistical Parametric Mapping (SPM) software implemented in Matlab (SPM8; Wellcome Department of Cognitive Neurology) and the FSL(2) in a manner similar to that of our prior publications.(3, 4) Briefly, motion correction was performed by realigning and unwarping the fMRI images to the first image of each task run after removal of the three dummy scans acquired at the start of the scanning session. Images were normalized to the stereotactic space of the Montreal Neurological Institute template.(5) T1-weighted data were normalized to standard space using the FMRIB nonlinear registration tool, and the functional echo-planar image data were co-registered to the T1 data using the FMRIB linear registration tool. Prior to computing brain activation values, physiological noise was estimated using the time series from an eroded mask within the ventricles and white matter and was removed from the motion-corrected fMRI time series. Functional data were then smoothed using an 8 mm Gaussian kernel and high-pass filtered using a cutoff period of 128 seconds.

Following realignment and unwarpinboxg, quality control diagnostics were completed on the time series data for each run. Quality control diagnostics included removing scans with incidental findings, scanner artefacts and signal dropout. Participants’ data were included if no more than 20% (30/151) of time points were censored for frame-wise displacement or variance spikes. This resulted in total of n = 61 and n = 50 for the baseline and 16-week imaging sessions respectively.

Defining regions of interest

Our regions of interest for the negative affect circuit engaged by threat and sad were defined in our protocol(6-8) and pre-planned analytic plan was established in a prior systematic procedure validated with the same facial emotion task as used in the present ENGAGE-2 trial.(9) Primary target regions of interest were the subgenual ACC (sgACC) and amygdala (bilaterally) for threat and the pregenual ACC (pgACC), amygdala (bilaterally), and anterior insula (bilaterally) for sad. Functional connectivity between ROIs and a global circuit dysfunction score for negative affect circuit engaged by threat in the non-conscious viewing condition and sad in the conscious viewing condition were also computed as the secondary neural targets. Other secondary neural targets included ROIs for the cognitive control circuit using the go-no go task, the default mode circuit, the negative affect circuit engaged by threat faces in the conscious viewing condition, and the positive affect circuit engaged by happy faces in the conscious viewing condition. These regions were defined *a priori* and not derived using a discovery analysis with the present ENGAGE-2 sample. Our *a priori* focus on these regions and pre-planned analytic strategy to test hypotheses, as outlined in the ENGAGE-2 protocol (8) was informed by our synthesis of the imaging findings for depression(6, 10) and prior trials in which imaging was included at the pre-trial baseline to predict outcomes for both behavioural and pharmacological interventions. We have demonstrated that masks to define these *a priori* regions are reliably generated using the meta-analytic platform Neurosynth.(11)

Specifically, the meta-analytic platform Neurosynth(11) with the search term “threat” was used to define the negative affective network. Analysis of Functional Neuroimaging’s (AFNI’s) 3dExtrema function was then used to identify peaks corresponding to our a priori regions of interest. Because some terms yielded maps with excessively large spatial extent, we imposed a restriction that each peak have a minimum z-score of 6 and each region extend no farther than 10mm from the peak. For the amygdala, Neurosynth maps were intersected with anatomically defined boundaries from the Automated Anatomical Labeling atlas.(12) Finally, all ROIs were intersected with each individual’s grey matter mask. Thus, each ROI was specific to the gray matter anatomy of each individual.

# Section F: Association between Digital Health Literacy and Depression Outcome

We evaluated the association between digital health literacy score and the HADS depression outcome (see **Figure S3**). The change in depression score (improvement) was greater for those with lower digital health literacy scores (range 1-4), in the treatment group.


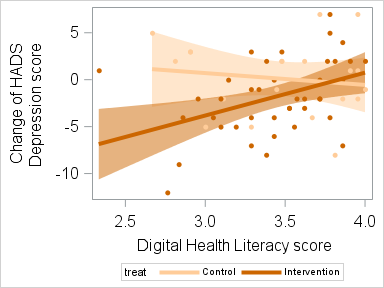


**Figure S2. Changes in HADS depression score with participants’ digital health literacy**

# Section G: Association of Neural Targets and Self-reports

The association between the considered regions of interest (ROI) and the self-reports also were also assessed. The primary ROIs were the amygdala (bilaterally) representing a key node in the negative affect circuit (for emotional reactivity), and the dorsal lateral prefrontal cortex (dlPFC) (bilaterally) (for the cognitive control circuit). The negative affect circuit was engaged by viewing of threat faces in the non-conscious viewing condition. The cognitive control circuit was engaged using a go-no go task.

For the emotional reactivity ROI (**Table S3**), there were no statistically significant associations between the considered self-reports (Positive Affect Score, Negative Affect Score and the Penn State Worry Questionnaire) in the treatment group. However, there was a statistically non-significant association between the Penn State Worry Questionnaire and the right Amygdala in the control group.

**Table S3. Correlations between changes in neural targets and changes in self-reported surveys at 16 weeks for emotional reactivity. *p<0.05; bolded values show r≥0.4 (positive or negative).**

| **Emotional reactivity** | **Overall (n=46)** | | | **Intervention (n=28)** | | | **Control (n=18)** | | |
| --- | --- | --- | --- | --- | --- | --- | --- | --- | --- |
|  | **Amygdala L** | **Amygdala R** | **Amygdala L** | | **Amygdala R** | **Amygdala L** | | **Amygdala R** |  |
| Positive Affect Score | 0.2 | 0.0 | 0.2 | | 0.0 | 0.2 | | 0.1 |  |
| Negative Affect Score | -0.1 | 0.1 | 0.0 | | 0.2 | -0.1 | | 0.0 |  |
| Penn State Worry Questionnaire | -0.1 | 0.0 | -0.2 | | -0.2 | 0.1 | | **0.4** |  |

The primary findings based on the right dlPFC circuit is presented in the main manuscript. The left dlPFC was positively correlated with an increased self-reported SPSI-R score indicating improved problem-solving ability (r=0.4, *p*=0.03), and negatively associated with the self-reported avoidant score indicating reduced avoidance (r=-0.5, *p*=0.002), from baseline to 16 weeks (this was similar to the findings on the right dlPFC) (**Table S4**).

**Table S4. Correlations between changes in neural targets and changes in self-reported surveys at 16 weeks for cognitive control. * p<0.05, bolded values show r≥0.4 (positive or negative).**

| **Cognitive control** | **Overall (n=47)** | | **Intervention (n=29)** | | **Control (n=18)** | |
| --- | --- | --- | --- | --- | --- | --- |
|  | **dlPFC L** | **dlPFC R** | **dlPFC L** | **dlPFC R** | **dlPFC L** | **dlPFC R** |
| SPSI-R:S raw score | 0.2 | 0.1 | **0.4*** | **0.4*** | -0.2 | **-0.5** |
| PPO raw score | 0.1 | 0.0 | 0.2 | 0.2 | -0.1 | -0.1 |
| NPO raw score | -0.1 | 0.0 | -0.2 | -0.3 | 0.0 | **0.4** |
| RPS raw score | 0.1 | 0.1 | 0.3 | 0.3 | 0.0 | -0.1 |
| ICS raw score | 0.1 | 0.0 | -0.1 | -0.3 | 0.3 | 0.3 |
| AS raw score | -0.3* | -0.1 | **-0.5*** | **-0.5*** | 0.1 | 0.3 |
| Dysfunctional Attitudes Scale | -0.2 | -0.2 | -0.3 | -0.1 | -0.1 | **-0.5*** |

The distribution for the changes in the self-reported SPSI-R score and the avoidant score (both from baseline to 16 weeks) with the changes in the right dlPFC circuit is shown in **Figure S4**.


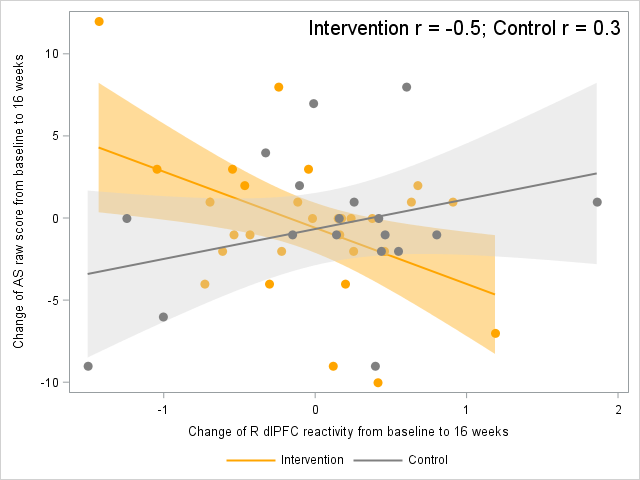

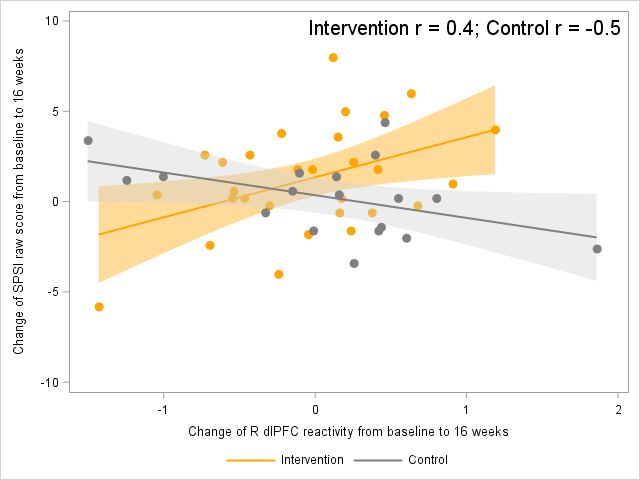


**Figure S3. Distribution of the changes in the right dlPFC circuit and changes in the SPSI-R score (left) and the avoidant score (right) from baseline to 16 weeks in the treatment and control groups.**

# Section H: Institutional Review Board approved Protocol

# Project Summary/Abstract

Depression and anxiety are the leading causes of disability and lost productivity, and are often underdiagnosed and undertreated owing to access, cost, and stigma barriers. Novel and scalable psychotherapies are urgently needed. Advances in artificial intelligence (AI) offer a transformative opportunity to develop intelligent voice assistants as virtual health agents accessible on personal devices. Meanwhile, major advances in human neuroscience have fueled a paradigm shift to study brain mechanisms underlying behavioral health interventions. Leveraging emerging science in these transdisciplinary areas, this project aims to develop and rigorously test a novel voice-enabled, AI virtual agent named Lumen, trained on Problem Solving Therapy (PST), for patients with moderate, untreated depressive and/or anxiety symptoms. The project will investigate the effect of Lumen on engagement of a priori neural targets—amygdala for emotional reactivity and dorsal lateral prefrontal cortex (DLPFC) for cognitive control—as putative mechanisms. The project has 2 phases: R61 and R33 (see Figure 1). Phase 1 is the R61 phase (years 1-2) and will be the sole focus of this protocol. Focusing on Lumen development and refinement as well as pilot testing, Phase 1 has 2 specific aims. Aim 1 on Lumen development and refinement will proceed in 2 stages: (1) scenario-based clinician evaluations, and (2) a formative user study (n=30 participants). Aim 2 is to pilot test Lumen in a 2-arm randomized clinical trial (pilot RCT), among 60 participants with moderate, untreated depression and/or anxiety who are randomized in a 2:1 ratio to receive PST with Lumen (n=40) on a secure study iPad or be on a waitlist (n=20). At weeks 0 and 16 participants will complete functional magnetic resonance imaging (fMRI) to assess neural target engagement as well as validated surveys of patient-reported outcomes (PROs) (e.g., depressive and anxiety symptoms, functioning, quality of life). In addition, participants will complete ecological daily assessments of mood, stress, appraisal and coping for 7 days every 2 weeks during the 16-week follow-up period. The Phase 1 milestones are (1) establishing the functionality, usability, and treatment fidelity of Lumen; and (2) demonstrating feasibility, acceptability, and neural target engagement. Achieving these milestones will provide the basis for the future R33 phase (Phase 2) focused on examining target engagement and PROs in a larger 3-arm RCT by comparing Lumen with a waitlist control arm and an in-person PST arm. This protocol only focuses on the R61 phase.

**The SPEAC Project**

**Phase 1:** **Developmental** **Research** (R61)

**Phase** **2: 3-arm RCT** (R33)

**Aim 2**

Pilot RCT (2-arm) with 60 participants to demonstrate **feasibility** and **acceptability** and test **neural target engagement**

**Aim 1**

Lumen design, development, and formative evaluation in **3 stages**

1. Scenario-based evaluations
2. Formative user study

**Pilot RCT**

RCT (3-arm) with 200 participants

Figure 1. SPEAC Project Overview

# Background/Scientific Rationale

Depression and anxiety cost an estimated $201 billion annually in the United States (US).(13, 14) Approximately 16 million US adults (6.7% of the population) experience major depression and 40 million (18.1%) experience anxiety disorders,(15, 16) and these disorders are often comorbid.(17) Moreover, subclinical symptomology not meeting the diagnostic criteria for major depression or anxiety is similarly common and is also associated with high disability and disease burden.(18-20) These conditions often go undiagnosed and untreated. Alarmingly, half of Americans with depression and 63% of those with anxiety do not receive any treatment, with the lowest rates among racial/ethnic minorities and persons of low socioeconomic status.(17, 21-23) People with mental illness often prefer psychotherapy to medication.(24-26) Yet, the reach and adoption of proven psychotherapies in mental health or general medical settings are limited, owing to barriers such as low reimbursement, provider shortage, patients’ lack of time and transportation, and stigma.(27-30) As such, there is a critically unmet need for empirically validated psychotherapies that have low cost, avoid stigma, and can be scaled to help address public health and health equity.

Technology-based interventions have quickly grown as a viable option for treatment delivery to address cost, access, and stigma barriers for traditional mental health services. The general public is highly receptive (76% reporting interest) to mental health monitoring and counseling using internet and mobile technologies,(31) and individuals with mental health concerns often prefer to seek help online rather than in person.(32) Studies have tested a broad range of technologies for delivering bona fide psychotherapies.(33, 34) The strength of the evidence on early-generation technology interventions led to depression and anxiety treatment guidelines recommending computerized cognitive behavioral therapies using web-based or interactive voice response systems.(35) Digital mental health interventions have since been evolving with technological advances. Several meta-analyses have documented growing evidence on the utility of mobile interventions for the management of depression and anxiety.(36, 37) One latest area of prolific technological growth in AI is around voice-based personal assistants, which are now nearly ubiquitous in personal home or mobile devices—with recent reports(38, 39) describing that worldwide sales of Alexa devices crossing 100 million and that of Google Home devices crossing 50 million.

With the advances in AI technology, creating persuasive systems that mimic human-like behaviors has become a realistic endeavor.(40, 41) The acceptability of AI-based systems in pragmatic applications is bolstered by studies that have shown that people are more willing to disclose personal information to a virtual agent than when they believe it is human-operated.(42) Empirical and theoretical research in the field of human-computer interface has shown that people anthropomorphize computers and complex technology(43, 44) and form social attitudes and behaviors and emotional responses towards them. Experiments showed conversational partners whether perceived to be text-based chatbots or humans were equally effective at creating emotional, relational, and psychological benefits,(45) and establishing strong therapeutic alliances.(46) Importantly, older adults and people with low reading and/or computer literacy find virtual agents approachable and usable,(47, 48) making these tools particularly relevant for addressing mental health disparities. Much of the development efforts to date have been centered around *text-based* conversational agents,(49) which have been shown to be feasible in a variety of settings,(50, 51) and for depression and anxiety counseling.(52) Although promising, text-based intelligent conversational agents have limitations including a lack of personalized interaction and difficulty for use among older adults or people with low literacy.(49, 53) In contrast, AI-powered personal assistants in dedicated voice devices (e.g., Amazon Echo, Google Home) and voice assistant applications (e.g., Alexa, Google Assistant, Siri) have advanced features to engage in human-like conversations. By utilizing automatic speech recognition, natural language processing, and deep learning algorithms, these voice assistants can be transformed from performing routine tasks (e.g., reporting current weather) to more sophisticated and context-specific health intervention tools. Research to develop and test such interventions is in its infancy. The focus of this protocol is to develop a *voice-based* AI agent that has both pragmatic viability and therapeutic utility for PST.

The *Study of a PST-Trained Voice-Enabled Artificial Intelligence Counselor (****SPEAC****) for Adults with Emotional Distress* protocol is designed to enhance and rigorously test an already prototyped, voice-based AI agent, **Lumen**. In accordance to the Lumen design and training plan (Appendix 1), a mature product will be developed through iterative, user-centered design-evaluation cycles based on the current prototype. The project will test the effect of Lumen on engaging empirically supported neural targets as well as the relationship of change in neural targets to change in PROs using validated surveys and ecological assessments (in natural living). This project addresses the National Institute of Mental Health’s strategic research priorities(54) and recommendations for developing novel information technologies in behavioral and social science research.(55) The public health impact lies in the potential to meaningfully improve the reach and impact of psychotherapy, mitigating access, cost, and stigma barriers for people with depression and/or anxiety who cannot or will not seek traditional mental health services or who desire more personalized, connected care. This may be particularly relevant to medically underserved populations (the target population in this study), thereby addressing health disparities.

**3.0 Objectives/Aims**

In Phase 1 of the SPEAC project, the specific aims are to: (1) establish the functionality, usability, and treatment fidelity of Lumen using iterative, user-centered design, development, and formative evaluation; and (2) demonstrate feasibility, acceptability, and target engagement in a 2-arm pilot RCT.

Aim 1: Formative Research

To achieve aim 1, we will conduct (1) scenario-based evaluations of fidelity by 2 PST master trainers; and (2) a formative user study with 30 eligible patients with depression and/or anxiety (defined below) who complete PST sessions with Lumen. The objective of the scenario-based evaluations is for 2 PST experts to assess the treatment fidelity of Lumen, and the completeness, consistency, and quality of the solution plan, and perceptions of Lumen. The objective of the formative user study is to test participant interaction with Lumen (e.g., what worked, what did not work, breakdowns in the interaction, understanding of developed solution implementation plan, perceptions regarding the viability of their plan, and general comments regarding the interaction) in order to evaluate usability,(56) user experience,(57) and therapeutic alliance(58-61) and complete Lumen development according to the Lumen design and training plan (Appendix 1).

Aim 2: Pilot RCT

To achieve aim 2, we will randomize 60 patients who score 10-19 on the Patient Health Questionnaire-9 (PHQ9) and/or 10-14 on the Generalized Anxiety Disorder-7 (GAD7), and randomize them in a 2:1 ratio to the Lumen treatment arm (n=40) or the waitlist control arm (n=20) for 16 weeks. The objectives of the pilot RCT are to determine feasibility and acceptability of Lumen as well as neural target engagement. Feasibility is assessed by being able to randomize 60 participants and retain ≥85% at week 16. Acceptability is assessed by having ≥95% Lumen participants complete≥1 PST session and ≥80% complete ≥4 sessions. To determine neural target engagement, the requirements are that both (a) the neural target for either nonconscious threat-related reactivity (amygdala) and/or cognitive control (DLPFC) improves meaningfully from week 0 to 16 for Lumen vs. waitlist control; and (b) change in either neural target significantly correlates with changes from weeks 0 to 16 in validated self-report measures corresponding with respective PST theory-based constructs for emotional reactivity or cognitive control.

**4.0 Eligibility**

Aim 1: Formative Research

The scenario-based evaluations are conducted by 2 independently contracted PST master trainers. The formative user study includes ENGAGE-2 (intervention and control) participants (n=30) screened for access to Lumen compatible Internet-enabled devices (iPad and/or smartphone.)

Aim 2: Pilot RCT

Racially/ethnically and socioeconomically diverse patients with moderate, untreated depression and/or anxiety symptoms who meet the eligibility criteria are included. Study participants are recruited from UI Health outpatient clinics. Eligibility criteria are detailed in Section 4.1 and 4.2. Participant eligibility is confirmed according to a multistep enrollment process detailed in Section 5. Trained research staff will assess eligibility per protocol and document findings in the REDCap (Research Electronic Data Capture) study database.

**4.1 Inclusion Criteria**

- Age: ≥ 18 years
- Emotional distress defined by elevated depressive (PHQ9 scores 10-19) and/or anxious symptoms (GAD7 scores 10-14)
- Willing and able to provide informed eConsent and HIPAA authorization

**4.2 Exclusion Criteria**

- Unable to speak, read, or understand English for informed consent
- Current pharmacotherapy or psychotherapy (individual or professionally led group therapy) for depression or anxiety (note: participants are not withdrawn post-randomization if they begin pharmacotherapy drugs or start psychotherapy during the study.)
- Suicidal ideation per PHQ9 with active plan
- Bipolar or psychotic disorder, or current psychiatric treatment
- Weight ≥325 pounds due to brain scanner constraints, MRI contraindications, traumatic brain injuries, and tumor or any other known structural abnormality in the brain (Aim 2 pilot RCT only)
- Severe medical condition (e.g., myocardial infarction or stroke or new cancer diagnosis in the past 6 months, end-stage organ failure, terminal illness) or residence in a long-term care facility
- Diagnosis of cancer (other than non-melanoma skin cancer) that is/was active or treated with radiation or chemotherapy within the past year
- Active alcohol or substance use disorder (including prescription drugs) based on the CAGE Questionnaire Adapted to Include Drugs (CAGE-AID)
- Cognitive impairment based on the Callahan 6-item screener
- Current or planned pregnancy or lactating (<6 months postpartum)
- Participation in other investigational treatment studies that would significantly affect participation in this study, raise safety concerns, and/or confound outcomes (participant may be asked to provide the informed consent of the other study for final decision on exclusion by a study psychiatrist)
- Family/household member of an already enrolled participant or of a study team member
- Plan to move out of the Chicago area during the study period
- Does not have reliable Wi-Fi Internet at home
- Unwillingness to user personal mobile device to receive study text messages
- Investigator discretion for clinical safety or protocol adherence reasons

**4.3 Excluded or Vulnerable Populations**

Children are excluded from the study as the target age group is 18 years and older. Psychotherapies of depression and anxiety and related risk protections (including protection of patient privacy and confidentiality) for persons under the age of 18 years differ from those for adults. Adults are the focus in this stage of research to develop a neural mechanism-validated, voice-based AI agent for PST. In addition, participants unable to speak, read and understand English for informed consent are excluded from the study as the research instruments and intervention are administered in English only.

**5.0 Subject Enrollment**

The procedures for participant recruitment, screening, and teleorientation with eConsent are the same for participants in Aim 1 formative user study and Aim 2 RCT, except where noted below. Subject enrollment follows a multi-step process as follows:

**Prescreening.** Aim 2: Pilot RCT only.

The primary recruitment strategy uses UI Health’s EHR database to identify patients meeting the basic prescreening criteria (e.g., age, absence of exclusionary medical or psychiatric comorbidities, etc.). Primary care providers (PCPs) have the option to review and identify patients who may be inappropriate for the study because of serious medical or psychiatric illness before recruitment invitations are sent. The secondary recruitment strategy uses in-clinic referrals or passive recruitment using advertising brochures and flyers at the UI Health outpatient clinics. Study brochures are distributed in clinic, and providers may refer patients during routine office visits and direct interested patients to the study website or telephone/text line for more information and screening. In addition, UIC listservs are used to distribute recruitment announcements to UIC employees. Based on previous experience using these recruitment strategies, it is anticipated that EHR-based recruitment will be the predominant source, accounting for >95% of enrolled participants. Reviews of patient EHR may be performed to screen for diagnostic exclusion criteria. This step is permissible under a waiver of informed consent. A waiver of informed consent and HIPAA preparatory to research are being requested for this process (medical record screening is minimal risk, will not affect patients’ rights or welfare, and without it, subject recruitment would be impracticable).

**Recruitment and Screening.**

Recruitment invitations are sent to patients by email, if an email address is available in EHR, or by texting a link to the “open for recruitment” announcement on the study website. The recruitment email and online announcement describe the study in lay language and contain a secure web link to REDCap where patients may give online consent to screen for eligibility, if interested, or decline further contact if they choose to opt out. Patients may also opt out by directly replying to the invitation email or text or by calling the study recruitment phone line.

For the Formative User Study patients will receive recruitment invitations (via email and/or text) up to three (3) times and will not be contacted via the phone if no response is received. For the Pilot RCT, patients who do not self-screen or opt-out within 2 weeks of the recruitment invitation send date, a trained study coordinator calls to assess interest and conduct screening by phone. Based on previous experience, it is anticipated that the ratio of patients screened eligible to patients fully eligible and randomized to be around 5:2 (in screening new patients for the RCT). A participant tracking database will be developed in REDCap, which supports Microsoft Excel exports for analysis and reporting on enrollment and screen failures at each step.

During initial eligibility screening participants, who score 10 or above on GAD-7 and /or PHQ-9 surveys of recent symptoms of depression or anxiety, are offered the option to receive resources for emotional health services via email, if interested.

**Teleorientation with eConsent**. Screened eligible participants are invited to schedule a teleorientation session with a study coordinator via their choice of telephone or online video conference. Teleorientation begins with the coordinator providing the REDCap link to the online consent document and leading the informed consent discussion, as outlined in Section 12, including study procedures, risks and benefits, the voluntary nature of the research, and the pros and cons of being randomly assigned to Intervention and waitlist control groups (pilot RCT only). Willing participants document their consent electronically using the secure REDCap eConsent framework and receive a copy of their signed consent in PDF form directly through REDCap. The study copy is automatically archived in the REDCap file repository. For the pilot RCT, eConsent is followed by the coordinator completing the final eligibility interview questions (see Appendix 2 screening measures).

**Scheduling study procedures.**

Aim 1: Formative Research

**Formative User Study**. After teleorientation, eligible participants are scheduled to attend the formative user study; a total of 30 participants will be scheduled.

Aim 2: Pilot RCT

**Pilot RCT**. After teleorientation, RCT participants are scheduled for a baseline assessment visit.. During the baseline visit, a trained study coordinator performs fMRI screening and data acquisition and collects baseline data per standardized measurement protocols. Females of child-bearing age who indicate any possibility that they may be pregnant must take a point-of-care urine pregnancy test. At the end of the baseline visit participants receive instructions to complete the initial ecological daily assessment for the next 7 days. Following successful completion of the baseline measures, fully eligible, consented participants are randomized to either Group A (Lumen Intervention) or Group B (waitlist control), at a 2:1 allocation.

**6.0 Study Design and Procedures**

Aim 1: Formative Research

**Scenario-based evaluations** do not include human subjects. These evaluations are carried out by 2 independent PST master trainers as contracted members of the research team, each of whom will assume the role of patients and interact with Lumen to complete 8 scenarios of PST encounters. These scripted scenarios will be based on previously recorded encounters with live counselors to broadly exploit various aspects of PST. After each scenario, they will provide feedback on quality and appropriateness of their interactions with Lumen.

The **formative user study** includes 30 participants with low, medium, or high digital health literacy based on the Digital Health Literacy Instrument.(62) After consent is obtained qualified study staff, each participant will complete a full PST session with Lumen in up to each of four 1-hour sessions. In addition, participants will download the paired application on their smartphones to test notifications, surveys, and ecological daily assessments. The formative research version of the Lumen PST session utilizes mock scores of recent psychological symptoms (PHQ-9 and GAD-7). Participants will only complete user-experience assessments and provide open-ended qualitative feedback on the interactive demo. Designated members of the study team (e.g. a researcher and a Lumen developer) will unobtrusively observe and listen to participant interactions with Lumen and take observation notes regarding critical incidents during the interaction (e.g., breakdowns in the conversation) and technical issues that were encountered. The modality of these unobtrusive observations (e.g. Zoom if conducted remotely, or from behind a one-way mirror if conducted in-person) will be determined by following the latest COVID-19 safety guidelines and precautions. Post-completion, participants perform a retrospective cognitive walkthrough to verbally describe their interaction with Lumen with the researcher. Cognitive walkthrough methods are commonly used for design and help in ascertaining the “cognitive fit” between the system and its users.(63) Specific emphasis during the walkthrough will be on participant interaction with Lumen: what worked (and what did not), breakdowns in the interaction, understanding of developed solution implementation plan, perceptions regarding the viability of their plan, and general comments regarding the interaction. The researcher and developer may prompt participants with additional questions based on their observation notes. Following cognitive walkthroughs participants complete 3 user surveys: (1) NASA Task Load Index (NASA-TLX)(56) of user workload along with 6 dimensions of mental, physical, temporal, performance, effort and frustration, (2) User Experience Questionnaire-Short version (UEQ-S),(57) and (3) the Working Alliance Inventory adapted for digital interventions (WAI-Tech).(60, 61) Survey data is collected via REDCap. Lastly, the 2 PST master trainers will evaluate audiorecorded Lumen PST sessions on: (1) treatment fidelity based on the 7-item PST Adherence and Competence Scale (PST-PAC),(64, 65) (2) completeness, consistency, and quality of the solution plan, and (3) perceptions of Lumen.

Aim 2: Pilot RCT

The objective of the pilot RCT is to demonstrate (1) the feasibility of participant recruitment and retention, (2) participant acceptability of Lumen for PST, and (3) engagement of neural targets subserving emotional reactivity and cognitive control (i.e., target engagement).

**Randomization and blinding**. A designated staff person performs randomization using an online system that Dr. Ma and team published.(66) The system is a modern implementation of Pocock and Simon’s minimization, a covariate-adaptive method(67) to achieve better-than-chance marginal balance between study arms across multiple key baseline characteristics. Minimization accommodates a greater number of balancing covariates than does stratified randomization.(68) The system’s computational algorithm automatically adjusts the randomization probability based on the characteristics of all the previously randomized participants, thus minimizing the total covariate imbalance between arms after each new participant is randomized. In this study, randomization covariates include sex, age, race/ethnicity, education, digital health literacy,(62) PHQ9, and GAD7. Note that, aside from eligibility screening, PHQ9 and GAD7 are also process measures used to monitor treatment progress throughout PST sessions. Their inclusion among randomization covariates helps avoid accidental bias at baseline. The online system applies Efron’s biased-coin method(69) to protect allocation concealment with the use of nonextreme randomization probabilities, and the staff person(s) using the system has/have no deep knowledge of the backend coding or execution and no ability to manipulate it. By design, treatment assignments are identifiable to participants, but blinding of outcome assessment, event adjudication, and data analysis will be enforced. To accomplish the 2:1 allocation, participants are randomized into 3 sets, and then 2 of these sets will be combined to form the Lumen group. The remaining set forms the waitlist control group. This method both protects blinding and preserves the allocation ratio at every allocation as per Kuznetsova and Tymofyeyev.(70) The same method is used in the ongoing ENGAGE-2 study.(71)

**Assessments**. All participants complete two study assessment visits, at baseline (week 0) and at 16 weeks, and self-complete ecological daily assessments for 7 days every 2 weeks. The fMRI scan visits are conducted at the 3T MR Research Program located at the UIMC Advanced Imaging Center, a BioRAFT registered facility, and SPEAC study staff work in compliance with all COVID-19 safety guidelines employed by facility management. When scheduling assessment visits, study coordinators instruct participants in the current COVID-19 safety precautions prior to and at the visits.

Assessments(120 minutes)

- fMRI assesses neural target engagement and treatment outcomes. fMRI is a standard technique for measuring and mapping brain activity that is noninvasive and safe. In this study, it is being used simply to gather neuro imaging data, and not to test the safety/efficacy of a device or software. Afterwards, the patient completes blood pressure, height, and weight measurements. (90 minutes)
- Self-report surveys of PST theory-based constructs of emotion (affect, worry) and cognition (problem solving, dysfunctional attitudes) as well as patient outcomes (e.g., depressive and anxiety symptoms, functioning, quality of life). Participants may complete these surveys during or outside their fMRI visits. (30 minutes).

Self-completed Ecological Daily Assessments (<3 minutes each day)

Using the Lumen companion application participants complete ecological end-of-day assessments of mood, stress, appraisal, and coping for 7 days every 2 weeks (over 16 weeks).

**Intervention Orientation**. Participants in the intervention arm attend a Lumen orientation visit (60 minutes) during which they will be given a Lumen intervention tutorial and receive a study iPad, configured to limit access to only the Lumen intervention enabled on the device. Before leaving participants are scheduled for the first PST session with Coach Lumen. As needed, Lumen intervention orientations can be done remotely.

**Intervention data collection.** Secure study iPads used by participants in the intervention arm are enabled with Lumen PST. Participants complete 8 PST sessions beginning with 4 weekly and then 4 biweekly intervals over 12 weeks on their assigned iPad. At the end of each PST session, participants are prompted to schedule their next session with Coach Lumen. They receive automated reminder notifications 1 day prior to their next session and on the session day and have the opportunity to make up missed sessions. Intervention participants also complete the PHQ9 and GAD7 and user experience surveys at all PST sessions. Upon completion of the intervention participants will receive a certificate of completion signed by Dr. Jun Ma. They may also complete a post-intervention interview via Zoom to characterize participant perspectives regarding their Lumen use experience.

**Intervention fidelity assurance.** All PST sessions that participants complete with Lumen are audiorecorded. Session recordings and transcripts are stored on the Amazon Web Services (AWS) in study configured accounts, with coded identifiers (see Section 7.). These materials will be used to evaluate treatment fidelity and user-Lumen interaction.

**7.0 Expected Risks/Benefits**

This is a minimal risk study with prudent measures in place to protect the health and well-being of research participants and their privacy and confidentiality. Risks associated with participation in this study may include the potential for the following:

- Potential for self-harm
- MRI-related injury, discomfort, and distress (Aim 2: pilot RCT only)
- Patient privacy and confidentiality breach
- Accidental recording.

These risks are largely associated with the characteristics of the patient population to be studied and the procedures involved in the research. The target population includes patients with moderate, untreated depressive and/or anxiety symptoms. The risks are reasonable in relation to the anticipated benefits and are minimized by using procedures that are consistent with sound research designs and established research and clinical protocols. The following measures are implemented to minimize potential risks to participants in the study.

**Protection against risks of self-harm**. Some of the questions about depression, thoughts of death and other psychological symptoms and conditions as a part of study assessments may cause discomfort for some participants. However, in general the questions are not particularly intrusive or distressing, and stress is likely transient. In addition, participants are free to refuse to answer any questions. It is widely accepted that asking questions about thoughts of death or suicide does not lead to increased risk of suicide. Nevertheless, in the event that a patient is identified as being suicidal during the screening or follow-up phase of the study, the following self-harm protection protocol (adapted from the UIC IRB approved ENGAGE-2 study #2018-1174), is in place to immediately alert the study supervising psychiatrist to assess the patient’s suicidal thoughts by telephone, followed by notification of the participant’s PCP and appropriate clinical action if necessary. If a participant responds “1” (“several days”), “2” (“more than half the days”), or “3” (“nearly every day”) to the PHQ-9 question “Over the last 2 weeks, how often have you been bothered by thoughts that you would be better off dead or thoughts of hurting yourself in some way?”, we further assess the participant’s level of risk by asking “*Do you have a plan for how you would commit suicide?*” and then follow the protocol based on the assessed level of risk. Individuals reporting suicidal ideation on PHQ9 with an active plan at eligibility screening are excluded from participation. Nonetheless, the risk of emergent suicidality still exists after enrollment. Risk for suicide may be detected when a participant completes the PHQ9 at the beginning of each PST session with Lumen. Participants who score 1-3 (see above) on item 9 of the PHQ9, will be offered the option to call an emergency contact, the national 800-SUICIDE/800-273-TALK hotline, or 911 at the time of detection. This information is a pre-programmed script for Lumen. The supervising study psychiatrist, Dr. Ajilore, the intervention manager, Mrs. Ronneberg, receive immediate notifications, as outlined in the table below. The study psychiatrist calls the participant within 3-4 days to conduct an assessment, assess need for further referral, and discuss referral options depending on urgency, needs and preferences, available supports in place, insurance status and routine source of care. The study psychiatrist may refer the participant for immediate comprehensive evaluation (e.g., at a local emergency department), ambulatory psychiatric services, or community resources, with personal accompaniment depending on the evaluation outcome.

| **[If PHQ9 completed with SPEAC study staff by phone]** | |
| --- | --- |
| **If YES, participant has active plan for self-harm:**   - Explain to participant: I am concerned for your safety and therefore need to call for help right now. - Get participant's location - Stay on the phone with participant and use another phone to call 911.   [Script: "*I want to report a self-harm alert for a UI Health research participant who has just endorsed suicidal ideation to me (by phone) - I want to provide his/her name, location, and phone number (any relevant detail the pt provided.)"]*   - Send High-priority Page to on-call Study Physician.   CRC Note: You do NOT need participant's consent to call 911 if you feel there is a possibility of immediate risk of harm to self or others. | **If NO active plan or DECLINE TO STATE, explain to participant:**  I am not a clinician; however, our study has clinicians who speak with any participant who tells us they've been feeling this way recently. I will have a study doctor call you within the next 3-4 days. I would also like to give you 2 national helpline and 1 text line numbers that you may find helpful. All numbers are available 24 hours/7 days a week. We will work together to get you feeling better.  National Hopeline Network: 1-(800)-SUICIDE or 1-(800)-784-2433  National Suicide Prevention Lifeline: 1-(800) 273-TALK or 1-(800)-273-8255 Crisis Text line: text START to 741741 |
| **[If PHQ9 completed by Participant online ]**  [The following pop-up message appears if a participant responds “1” (“several days”), “2” (“more than half the days”), or “3” (“nearly every day”) to the 9th question, regardless of active action plan or not.]  Please note: We do not monitor this screener in real time, if this is an emergency call 911.  For more immediate attention, because you have been bothered by thoughts that you would be better off dead or of hurting yourself in some way in the last 2 weeks, you should call your physician or other healthcare professional right away or go to the emergency room.  You may also call the National Suicide Hotline at 800-SUICIDE / 800-784-2433 or the National Suicide Prevention Lifeline at 800-273-TALK / 800-273-8255. You may also text START to Crisis Text line number 741741. All these numbers are available 24 hours every day.  We will have a study doctor contact you within 3-4 days. In the meantime, do not delay seeking medical attention. | |

**Protection against injury, discomfort, and distress with brain imaging**. (Pilot RCT only)

MRI is non-invasive, widely used, and safe. Routine contraindications to MRI include presence of any metal implants (pacemaker, aneurysm clips, neurostimulators, cochlear, eye implants, old or very fresh tattoos). Participants are thoroughly assessed for MRI eligibility during screening using the well-established procedures as in the ENGAGE-2 study (protocol #2018-1174). A small number of people may feel claustrophobic inside the MRI machine. The study can be immediately stopped via button press if this occurs. Sometimes subjects report a temporary, slight dizziness or light-headedness when they come out of the scanner. Study personnel and technicians are on site during all acquisitions to address any such discomfort. To minimize fatigue, sufficient breaks are provided to participants during the scanning procedure, and the study coordinator conducting the scan regularly inquires as to whether there is anything that s/he can do to facilitate the participant’s comfort. In the event of adverse effects related to MRI scanning, study personnel and medical staff are on-site for consultation and assistance. The UI Hospital Emergency Room is less than a 5-minute drive from the scanning center. If there are any adverse events at any time during the MRI procedure, the study coordinator terminates the scanning session, provides a debriefing, and contacts study psychiatrist (Dr. Ajilore) for assistance and follow-up for the participant.

According to the National Institute of Mental Health (NIMH) Council Workgroup on MRI Research and Practices (September, 2005), “there is no known risk of MR brain scanning of a pregnant woman to the developing fetus for scanning at 4T or less, and no known mechanism of potential risks under normal operating procedures.” Notwithstanding, subjects are warned about potential risks not yet discovered in the informed consent form. Before each scan, female participants are asked if they are or are trying to become pregnant and when they had their last menstrual period. Any woman who indicates that she is pregnant will not be scanned. Any woman who indicates that she is trying to become pregnant or who is experiencing a late menstrual period is asked to complete a urine pregnancy test, which if positive will preclude the subject from being scanned.

If study personnel observe any unusual features in the MRI scan at the time of acquisition or an incidental finding, or abnormality on MRI scans, staff requests a clinical neuroradiological report on the scan from the neuroradiologists at the Center for MR Research who are on call to provide these reports as required, as part of the infrastructure of the Center’s facility. During the consenting process, all participants are informed about the potential risks of discovering an incidental finding or abnormality on their MRI scan. If an abnormality is found in a participant’s MRI scan, PI Dr. Ajilore contacts the participant and refers him/her for medical follow-up for the problem if the participant requests, including a referral to a PCP. If a participant has a PCP, the PI contacts the PCP, at the request of, and with verbal permission from the participant, to inform him/her of the finding on the MRI scan and to help him/her get the participant appropriate follow-up. The decision as to whether to proceed with further examination and/or treatment lies solely with the participant and his/her PCP.

**Protection against breaches of participant privacy and confidentiality**. All investigators and their staff are adequately trained to protect participant privacy and sign an agreement to do so. All information obtained from research participants during the study are considered strictly confidential and are only used and disclosed as permitted under the HIPAA regulations. All eligible participants must sign a HIPAA authorization as part of the informed consent form in order to participate. They also have the option to agree to sharing of their de-identified data through the NIMH Data Archive (NDA). Only aggregate data will be included in scientific presentations and publications resulting from this study.

To ensure data security, security protocols are incorporated at multiple levels. First, participants are assigned unique anonymous IDs (“Study Participant ID”) that are used as a proxy for mapping all PHI and PII (personally identifiable information). A mapping document that links Study Participant IDs to the corresponding PHI and PII is maintained separately from all study-related data of participants and stored in a secure manner (e.g., encrypted REDCap database on a secure server behind the university’s fire wall). Only a designated subset of study personnel who have a need to know (e.g., PIs and recruitment personnel) have access to the mapping document.

Aim 1: Formative Research

**Formative user study sessions**: present minimal risk to research participants and prudent measures are taken to protect their privacy and confidentiality. Participants are informed in advance about the discussion topics so that they may make an informed decision to participate. For the formative user study sessions, participants are made aware of study team observers present during the sessions. Study personnel alerts participants before beginning audio-recording of sessions. Transcriptions of audio-recordings do not identify participants, instead individuals are referred to as “Respondent #.” If names or locations of attendees are recorded during a session, that information will not be included in the transcripts. Federal regulations require research records be retained for at least 3 years after completion of the research.

Aim 2: Pilot RCT

In addition to the protection measures described above, randomized participants who begin intervention per group assignment, receive study a iPad which utilizes the in-built encryption and 6-digit passcode protection and is configured with their first name, (as per informed consent.) Only first name will be used by Coach Lumen during intervention to facilitate participant engagement for treatment alliance. Additionally, the study iPads are in a “lock down” mode where participants cannot use them for any purposes but for tasks associated with the study.

No study data resides locally on the iPad. In order to maintain anonymity of the intervention participants, no personal identifiers (other than first name) are used during Lumen PST session data acquisition or storage. All Lumen PST session recordings and transcripts acquired by the Amazon Alexa application using the study iPad are stored on the AWS cloud within study configured accounts. The study-established AWS accounts are assigned coded “AWS IDs” (which are paired with “Study Participant ID”) are accessible only by study staff with a need to know. In case of a potential loss or theft of the study iPad, participants are instructed to immediately notify study staff and the AWS ID account access will be disabled.

The SPEAC study is a registered HIPAA account under a standardized Business Associate Addendum (BAA) held by UIC/UI Health with (AWS) which enables the covered entities to be HIPAA compliant.

Finally, participants are able to keep their study iPad for personal use after their AWS ID account access is disabled. Participants will be provided with instructions to remove the Lumen skill and to unlock the iPad to have a fully enabled device to keep. Alternatively, participants may choose to redeem the iPad for $100. Participants in the waitlist control arm in the pilot RCT may choose to attend a Lumen Orientation visit to receive training and a Lumen PST-enabled iPad after the end-of-study assessments at 16 weeks post randomization. These options are explained to study participants during the informed consent and reiterated to waitlist control participants who choose to receive Lumen PST after the end of the study.

**Protection against accidental recording**. Accidental recording is a commonly raised concern regarding voice-enabled technologies. In the case of Lumen, the application is installed on the locked-down and encrypted study iPad. In order to prevent accidental recording, the following protections are implemented: First, the iPad has a keypad lock. Recording takes place only in the unlocked mode, after the participant opens the Alexa app, and gives permission verbally (using specific “wake phrases” such as “Launch Lumen Session,” or by tapping the option on the iPad screen. Second, participants are informed that all Lumen sessions are recorded and give explicit permission for this as part of their informed consent process. Third, if the study iPad is left unattended during an active session, the iPad locks after 10-seconds, and after 30 seconds of no verbal input from the participant, the Lumen session shuts down (preventing any further recording). Once the participant returns, they must go through the process of unlocking iPad and waking Lumen to actively resuming their session.

**Potential benefits to participants**. Eligible participants in the study have mild or moderate depressive and/or anxiety symptoms that are not treated. They receive PST by interacting with Lumen on a secure study iPad. Waitlist controls have the option to receive Lumen after their 16-week assessments. PST is a brief skill-enhancing psychotherapy with robust evidence for efficacy in treating depression and anxiety. PST focuses on improving one’s skills involved in solving personal real-life problems and is easy for most people to understand. Its stepwise, patient-driven approach is also easy to follow. Lumen is a voice-enabled, PST-trained agent who will undergo iterative design development and refinement based on information gathered from evaluation cycles and rigorous testing. Through interacting with Lumen, participants in the study may benefit from having PST at their fingertips and experience improvements in psychological symptoms, functioning and quality of life. If shown successful in this developmental R61 phase of the project, Lumen will undergo further confirmatory testing (in the R33 phase) and may downstream carry the potential to be scaled for use by people with depression and/or anxiety who cannot or will not seek professional help or who desire as-needed, personal help beyond what conventional treatment models can offer.

**8.0 Data Collection and Management Procedures**

Aim 1: Formative Research

**Scenario-Based Evaluations** by the 2 independent PST master trainers are completed and stored on REDCap at UIC.

**Formative User Study** data from audiorecorded Zoom conference session, with participant’s explicit permission.

Aim 2: Pilot RCT

The study coordinators are trained on data collection according to standardized protocols, and their performance is continuously monitored. The following types of data are collected.

1. Data on diagnoses, prescriptions, clinical encounters, and hospitalizations are abstracted from EHR for eligibility screening and baseline characterization.
2. fMRI data and physical measurements are collected at the UIMC Advanced Imaging Center.
3. Survey data and ecological daily assessments are collected using self- and interviewer-administered questionnaires on REDCap, a HIPAA-compliant server.
4. With participant’s explicit permission, PST sessions are audiorecorded and are stored in study configured accounts on the AWS cloud, accessible only by study staff.

See Appendix 2 for the list of study measures and assessment schedule.

**8.1 Data Management**

**Neuroimaging data management.** There are Linux machines dedicated to various aspects of image transfer and storage available to the MPI Dr. Ajilore and team within the UIC Psychiatric Institutes. The Institute also houses an imaging processing server for data storage and analysis (Dell PowerEdge R900, 2.13 GHz, Xenon Four Cores, 128GB RAM with 6 TB of data storage). These machines are networked with a T3 line to facilitate image transfer and back-up of data from the Center for MR Research. The T3 line is connected to the University network and firewall shielded from the outside. These machines are for the sole use of the research team and as such the research team has its own log in for the system with direct access from our dedicated research computers.

**Non-imaging data management.** REDCap study database on a HIPAA-compliant server, occur automatically according to a preset schedule (no participant action required). Data are immediately available for inspection and reporting of recruitment and retention status and any missing data. The data analyst performs weekly quality controls.

All datasets are cleaned, verified, and archived. One official copy of all study data and a master data dictionary are maintained and updated regularly. All analytic and tracking databases are stored on a HIPAA-compliant server with continuous backups. For the protection of participant confidentiality, unique anonymous study IDs are used for data storing, tracking, and reporting. PHI is stored separately from all other study data and will be used and disclosed in accordance with the HIPAA regulations. Regular reports are produced on (1) patient accrual and follow-up completion/retention in relation to goals and timeline; (2) the randomization process and group comparability on the balancing variables; (3) key baseline characteristics of the sample, by (blinded) group, related to the primary and secondary outcome variables; (4) intervention exposure and adherence; and (5) protocol violations. Any observed delays in these processes or data irregularities shall be followed up and resolved in a timely manner.

**Data sharing.** De-identified fMRI data and other study data are shared with collaborators at UIC and other collaborating institutions via a UIC Box Health Data Folder. All collaborating institutions are listed as data users under the HIPAA regulations and authorization provided by participants. PHI (e.g., name, address, phone number) and the link between study ID and the patients’ identities will not be shared. They can download data from the UIC Box Health Data Folder and store them on a HIPAA-compliant server at their institution for analysis according to the study protocol. We follow the latest industry standards for data encryption, server authentication, and client authentication to ensure secure data transmissions at all times. In addition, all the investigators and staff maintain up-to-date trainings and certifications in human subject’s protection, HIPAA, and Good Clinical Practice (GCP). Furthermore, we will partner with the NIMH and use all NIMH data preparation and sharing policies as a guide to ensure the deidentified data and results, along with all associated documentation, are submitted to the NIMH Data Archive (NDA), to be specifically deposited to the National Database for Clinical Trials Related to Mental Illness (NDCT). We will ensure we have permission from our participants to disclose deidentified participant-level data collected as part of this study to researchers who meet all the NDCT requirements for requesting use of the dataset. We will strictly comply with the HIPAA and IRB regulation requirements regarding research use of PHI and appropriate safeguards for sharing deidentified data.

**9.0 Data Analysis and Statistical Considerations**

Aim 1: Formative Research

**Formative User Study.** We will conduct the following analyses: (1) analysis of verbal interactions with Lumen to measure interaction efficiency, including breakdowns in conversations (e.g., stoppages) and pauses that break the flow and continuity, and consequently, the efficiency of the conversational interaction; (2) analysis of Digital Health Literacy Instrument,(62) task load (NASA Task Load Index (NASA-TLX)(56) of user workload along with 6 dimensions of mental, physical, temporal, performance, effort and frustration), user experience (User Experience Questionnaire-Short version (UEQ-S),)(57) and therapeutic alliance (Working Alliance Inventory adapted for digital interventions (WAI-Tech)(60, 61) and (3) content analysis(72) of audiorecordings of the walkthrough sessions. Quantitative survey data are summarized with descriptive statistics using SAS software (version 9.4; SAS Institute Inc.). Qualitative data are analyzed using the content analysis method descried above.

Aim 2: Pilot RCT

Pilot RCT sample size calculation using a confidence interval approach: To obtain a precision interval with a standardized half-width of 0.50 (akin to a medium effect) with 90% assurance, we have planned a sample size of 60 (n*_Trt_*=40, n*_Con_*=20), assuming ≥85% retention at 16 weeks. This provides reliable estimates of between-treatment differences in change in amygdala activation for nonconscious threat-related reactivity and change in DLPFC activation for cognitive control from week 0 to 16 in Criterion 1. In Criterion 2, a sample size of 60 will also be sufficient to detect a correlation coefficient of r=0.4 with 0.80 power and 2-sided α=0.05. We are not focused on smaller correlations (r<0.4) because their implications are likely to have more limited clinical relevance.

The criteria to evaluate neural target engagement are as follows:

***Criterion 1***. The level of task-evoked activation for either the amygdala for nonconscious threat-related emotional reactivity or for the DLPFC for cognitive control improves meaningfully from week 0 to 16 in the Lumen group compared with the waitlist control group.

Criterion 1 is tested via *t* test that compares the change from week 0 to 16 in the Lumen group with that change in the waitlist control group. Participants are analyzed based on the group to which they are assigned using all available data. The *t* test described above can be readily expanded for exploratory subgroup analyses by applying to each subgroup (e.g., female vs. male).

***Criterion 2***. Change in the level of task-evoked activation of a neural target that meets Criterion 1 correlates with temporally concurrent change from week 0 to 16 in self-reported measures within the corresponding PST theory-based construct for emotional reactivity or cognitive control. We consider correlations of r≥0.4 meaningful.

To test Criterion 2, we examine the regression change of the amygdala activity from week 0 to 16 on change in self-reported measures of emotional reactivity (worry, affect) from week 0 to 16, controlling for baseline values of the self-reported measures and group assignment. We expect that the coefficient for the change in amygdala activation is significant and positive in the direction of improvement in its corresponding, validated emotional reactivity self-report measures and that this relationship may be significantly modified by group assignment. Similarly, we examine the regression change of the DLPFC activity from week 0 to 16 on change in self-reported measures of cognitive control (problem solving, dysfunctional attitudes) from week 0 to 16, controlling for baseline values of the self-reported measures and group assignment. We expect that the coefficient for the change in DLPFC activation is significant and positive in the direction of improvement in its corresponding, cognitive control validated self-report measures and that this relationship may be significantly modified by group assignment.

In addition, we will also conduct exploratory and secondary analyses. We will conduct an exploratory whole-brain analysis (1) to verify our *a priori* specification of neural targets, namely, amygdala for emotional reactivity and DLPFC for cognitive control, and (2) to identify any additional voxel clusters that are activated by our tasks and are associated with treatment response. Secondary analyses compare differences in discrete changes in PROs from week 0 to 16 and trajectories of change based on intensive time-series ecological measures between the Lumen and waitlist control groups. Analyses of the treatment effects on PROs employ the same *t* test approach as described above for testing Criterion 1 for target engagement. Furthermore, dosing analyses are conducted within the Lumen treatment group. The primary dosing analysis examines the relationship of neural target activation in the amygdala and DLPFC to the 4 measures of dose (number and length of PST sessions, fidelity-weighted number, and length of PST sessions). The secondary dosing analysis examines the relationship of patient-report outcomes, ecological end-of-day assessments, and measures of treatment progress (e.g., PHQ9 and GAD7 across PST sessions) to the same 4 measures of dose. Given neural target activation measures and PROs are obtained at weeks 0 and 16, these analyses focus on change over the full 16-week span of the study, whereas ecological end-of-day assessments and treatment progress measures are available for a total of 8 occasions each, and thus will be considered longitudinally.

Please see Appendix 3 for detailed power analysis and statistical analysis plan.

**10.0 Quality Control and Quality Assurance**

REDCap built-in quality control features (e.g., data type validation, valid values/range rules, required responses) and extensive data rules are used to allow for complex real-time quality control (QC) checks (e.g., of missing values, out of range values, logic comparisons, cross form/event consistency). When issues are identified through weekly and monthly scheduled QC checks, the data analyst will use the “REDCap data resolution workflow” feature, a built-in data query tool, to communicate the issues to study coordinators for resolution. Study coordinators can add notes or corrections within the data query tool and have editing rights to alter the source data, but all edits must be approved by the project manager. REDCap tracks all correspondence and decisions of the query process, and it maintains an extensive audit trail of access, entry, and edits of stored data. REDCap also allows for form and record locking capability with e-signature management.

**11.0 Data and Safety Monitoring**

The following Data and Safety Monitoring Plan (DSMP) will be followed to ensure the safety of study participants and the validity and integrity of data in compliance with NIMH requirements.

**Independent Oversight.** Because the risks for adverse events (AEs) and data breaches are minimal in the pilot RCT, and the study is not a Phase III clinical trial, it is determined that a Data and Safety Monitoring Board (DSMB) is not needed. Instead a safety monitor *independent of* the study is responsible for overseeing the implementation of this DSMP to ensure (1) the protection and safety of human subjects and (2) the validity and integrity of the trial. Dr. Bernice Man, MD, o at the University of Illinois at Chicago (UIC), serves in this role. The safety officer is responsible for examining aggregate data on recruitment and retention, adverse events (unexpected or serious), and subject complaints, but he will not be reviewing individual, identifiable, subject data. If unexpected or serious adverse events occur, these will be reported to the IRB in the form of Prompt Reports. See further details below.

Contact PI Dr. Ma and senior biostatistician Dr. Xiao, both of whom will be blinded during the pilot RCT, meet with Dr. Man every 6 months during active participant recruitment and follow-up to present and discuss data and safety monitoring information (below). For the annual continuing renewal application to the IRB and the annual progress report to the sponsor each year, Dr. Man will provide a summary of her findings and recommendations regarding the following:

- ascertainment and any actions to be taken in response to AEs and SAEs reported during the study
- reports related to study operations and the quality of the data
- possible modifications in the study protocol concerning recruitment, participant retention, data quality, or trial operations more generally.

**Safety Monitoring.** As in any clinical trial, it is not possible to anticipate all possible AEs. Staff will undergo extensive training in ascertaining, monitoring, and documenting AEs—serious or not. The study investigators have extensive experience in clinical trial organization and management, including data and safety monitoring for single site and multisite trials. Established procedures for rendering first aid and life-threatening emergencies will be monitored by Dr. Ajilore (psychiatrist).

An AE is defined as any untoward medical or psychological event experienced by a patient during or as a result of his/her participation in the study that represents a new symptom or an exacerbation of an existing condition whether or not considered study-related based on appropriate medical judgment. SAEs are any adverse experience that results in any of the following outcomes:

- Death
- Life-threatening event/illness
- Inpatient hospitalization or prolongation of existing hospitalization
- Persistent or significant disability/incapacity
- Pregnancy resulting in a congenital anomaly/birth defect
- Any event requiring medical or surgical intervention to prevent permanent impairment or damage. In this study, this is defined as physician confirmed diagnosis of any of the following: angina pectoris, heart attack, stroke, transient ischemic attack, heart failure, coronary angioplasty or bypass surgery, peripheral vascular disease, , any other serious injury to the bone or muscle, liver failure, kidney failure, and cancer (except for non-melanoma skin cancer).

Non-serious AEs are all adverse events that do not meet the above criteria for “serious.” To ensure unbiased ascertainment, AEs will be systematically identified by querying participants at 16-week follow-up visits using the AE Patient Query Form. In order to access the electronic medical record, as needed, for AE adjudication, consenting participants are asked to provide and/or confirm the minimum necessary identifiers including first and last name, date of birth, mailing address, and whether they have received care at UI Health.

Blinded Reporting. In the pilot RCT, safety information will be monitored while keeping the true identity of the study groups masked. In his role as the study physician, Dr. Ajilore may need to become unblinded. But Contact PI Dr. Ma and biostatistician Dr. Xiao as well as the independent safety monitor Dr. Man will be blinded throughout the study and will review summaries of the numbers and rates of all AEs by blinded treatment group. Proper blinding of the other investigators and outcome assessors will be enforced as well.

Requirements for Adverse Event reporting. The Contact PI , Dr. Ma, will inform the Director of IRB panel and all relevant oversight committees at the university within 5 business days of learning of an unanticipated AE or major protocol deviation. All relevant information will be reported to the IRB for each unexpected SAE including information about the event and its outcome, dosing history of a suspect medication/treatment, concomitant medications, the participant’s medical history and current conditions, and all relevant laboratory data. Within 15 business days of the PIs becoming aware of changes in risk/benefit or events requiring report to the sponsor, these will be reported. This timeline satisfies the NIMH reporting requirements for AEs and unanticipated problems. An annual report will be submitted to the IRB and to the sponsor summarizing all AEs, serious or not.

**12.0 Regulatory Requirements**

**Role of Collaborating Institutions in the Oversight and Conduct of Human Subjects Research.** The proposed study is a collaboration between UIC (primary institution), Pennsylvania State University (PSU), Washington University in St. Louis (WashU), and Stanford University. All study participants are patients of UI Health, and the UIC IRB is the IRB of record. Dr. Jun Ma, MD, PhD, and Dr. Olusola Ajilore, MD, PhD, both of UIC are co-principal investigators. Also, at UIC, Dr. Philip Yu, PhD brings expertise on machine learning and AI development, and Dr, Ben Gerber, MD, MPH provides expertise on implementation and evaluation of digital health behavioral interventions in disadvantaged populations of diverse racial and ethnic backgrounds. As the Study Physician, Dr. Ajilore (psychiatrist) oversees the clinical aspects of the study, involving patient recruitment and safety, and as needed, coordinate care with participants’ PCP. For more details, see Section 7. Dr. Joshua Smyth, PhD at PSU is a renowned expert in stress resilience and recovery and ecological momentary assessment (EMA). Dr. Smyth collaborates with the UIC team and is responsible for the PSU research team. Dr. Thomas Kannampallil, PhD at WashU brings expertise on Lumen design and development, human-computer interaction, and bioinformatics. Dr. Lan Xiao, PhD at Stanford is an experienced biostatistician and is responsible for developing and executing the statistical analysis plan of this project.

Informed Consent. All study participants involved in both the formative research and the pilot RCT must provide eConsent. The eConsent is obtained remotely via telephone or online video conferenced teleorientation session with trained study staff who leads the participant through the consent process and answer any questions about the study. Study personnel obtaining informed consent are experienced in obtaining informed consent and receive standardized training in trial-specific protocols. Risks and benefits and the voluntary nature of the study will be thoroughly explained by the study personnel. The participant will have as much time and information as needed to consider whether or not to participate.

Aim 1: Formative Research

ENGAGE-2 participants (enrolled but not active in the 2018-1174 protocol) who first screen eligible for access to Lumen compatible Internet-enabled devices, are scheduled for teleorientation with study staff who lead a full informed consent discussion and use the eConsent framework to provide written informed consent documentation (ICD).

Aim 2: Pilot RCT

The study uses a 2-step consent process, an online screening consent is first obtained prior to initial eligibility screening, which facilitates participant self-screening. Second, screened eligible and interested participants are invited to attend a teleorientation with study staff who lead a full informed consent discussion and use the eConsent framework to provide written informed consent documentation (ICD).

All participants document their consent electronically using the secure REDCap eConsent framework and obtain their pdf copy of the signed consent by directly downloading through REDCap. The study copy is automatically archived in the file repository in REDCap database, which is a HIPAA-compliant server, accessible to the PIs and study personnel.

**Subject Confidentiality.** See Protection against breaches of participant privacy and confidentiality in Section 7.0.

**Unanticipated Problems.** An unanticipated problem (UP) is defined to include any incident, experience, or outcome that meets all of the following criteria:

* Unexpected (in terms of nature, severity, or frequency) given (a) the research procedures that are described in the protocol-related documents, such as the IRB-approved research protocol and informed consent document; and (b) the expected natural progression of any underlying disease, disorder, or condition of the subject experiencing the UP and the subject’s predisposing risk factor profile for the UP;

* At least possibly related to participation in the research (possibly related means there is a reasonable possibility that the incident, experience, or outcome may have been caused by the procedures involved in the research); and

* Suggests that the research places subjects or others at a greater risk of harm (including physical, psychological, economic, or social harm) than was previously known or recognized.

Within 5 business days of the PI learning of a UP or major protocol deviation, the PI informs the Director of IRB panel and all relevant oversight committees at the university. Within 15 business days of the PI becoming aware of non-serious AE, changes in risk/benefit, or events requiring report to the sponsor, these will be reported. An annual report will be submitted to the IRB and the sponsor summarizing all AEs, serious or not.

**Appendices**

**Appendix 2. SPEAC Schedule of Measures – Aim 2 Pilot RCT**

| **Measure** | **Instrument** | **Collection Method** |  | **Time** (weeks)**^a^** | | |
| --- | --- | --- | --- | --- | --- | --- |
|  |  |  | **Screening** | **0** | **16** | **1-12** |
| Eligibility criteria | Age, sex, current/planned pregnancy/lactation, etc. | Self-report | **X** |  |  |  |
| Depression severity | Patient Health Questionnaire-9 (PHQ9), suicidal ideation (item #9) | Self-report | **X** |  |  |  |
| Anxiety | Generalized Anxiety Disorder Scale (GAD7) | Self-report | **X** |  |  |  |
| Alcohol/substance abuse | CAGE Adapted to Include Drugs (CAGE-AID) | Self-report | **X** |  |  |  |
| Brain scan screening | Brain scan screening questions (e.g., self-reported weight) | Self-report | **X** |  |  |  |
| Cognitive Impairment | Callahan 6-item screener | Interview | **X** |  |  |  |
| Emotional reactivity and cognitive control | fMRI | Measured |  | **X** | **X** | NA |
| Emotional reactivity and cognitive control | Penn State Worry Questionnaire(73)  Positive and Negative Affect Schedule (PANAS)(74) | self-report |  | **X** | **X** | NA |
|  | Social Problem Solving Inventory-Revised: Short (SPSI-R:S)(75)  Dysfunctional Attitudes Scale(76) | self-report |  | **X** | **X** | NA |
| Depression and anxiety symptoms | Hospital Anxiety and Depression Scale (HADS)(77, 78) | self-report |  | **X^b^** | **X^b^** | NA |
| Functioning | Sheehan Disability Scale,(79) Work productivity and activity impairment questionnaire (WPAI)(80, 81) | self-report |  | **X** | **X** | NA |
| Quality of life | 12-item Short-Form Health Survey (SF12)(82) | self-report |  | **X** | **X** | NA |
| Mood | Daily mood (items from circumplex model, capturing affective valence and arousal) | Ecological end-of-day assessments, per Event Window table in Appendix 4. |  | **X** | **X** | **X^c^** |
| Stress | Daily stress events (15 categories), perceived stressor severity, stressor-related thoughts |  |  | **X** | **X** | **X^c^** |
| Appraisal | Assessing daily implementation of “problem orientation” (challenge appraisals, optimism, self-efficacy, outcome expectancies) |  |  | **X** | **X** | **X^c^** |
| Coping | Assessing daily implementation of “problem solving-style” (attempts to understand problems, plan effective solutions to coping, impulsivity, carelessness, avoidance behaviors) |  |  | **X** | **X** | **X^c^** |
| Depression and anxiety symptoms | PHQ9 and GAD7 | Self-report and data tracking during PST sessions, per Event Window table in Appendix. 4 |  | **X^b^** | NA | **X^b^** |
| Treatment acceptability | Session metrics: Number and duration of PST sessions completed; End-of-session Lumen user survey: NASA Task Load Index (TLX),(56) User Experience Questionnaire-Short version (UEQ-S),(57) adapted Working Alliance Inventory for digital interventions (WAI-Tech)(60, 61) |  |  | NA | NA | **X** |
| Digital health literacy | Digital Health Literacy Instrument(62) | self-report |  | **X** | NA | NA |
| COVID impact | COVID impact survey(83) | self-report |  | **X** | NA | NA |
| Religious involvement | Duke University Religion Index(84) | Self-report |  | **X** | NA | NA |
| COVID impact on social functioning | COVID impact on social functioning survey | self-report |  | **X** | **X** | NA |
| Social network | Social Network Index(85, 86) | Self-report |  | NA | NA | **X** |
| Loneliness | UCLA 3-item loneliness survey(87) | Self-report |  | NA | NA | **X** |
| Height | Height | Measured |  | **X** | NA | NA |
| Weight | Body weight | Measured |  | **X** | **X** | NA |
| Blood pressure | Blood pressure | Measured |  | **X** | **X** | NA |
| Sociodemographics | age, sex, race, ethnicity, education, race, ethnicity, income, household size, marital status, employment status, occupation, smoking/vaping | self-report |  | **X** | NA | NA |
| Adverse Events (AE) | AE form | Interview |  | **X** | **X** | NA |

**^a^**To minimize missing data, participants will receive $50 at wk 0, study iPad (unlocked) or $100 (if opting out iPad) at wk 16, and $1 per daily diary.

**^b^**HADS is used as the independent outcome measure of depressive and anxiety symptoms at weeks 0 and 16. PHQ9 and GAD7 are used for 2 purposes: (1) eligibility screening (before week 0) and (2) treatment progress monitoring across PST sessions (in weeks 1, 2, 3, 4, 6, 8, 10, 12).

**^c^**In addition to ecological daily assessments that will occur for 7 days at weeks 0 and 16, they also will occur every 2 weeks (on weeks 2, 4, 6, 8, 10, 12). During active treatment in the Lumen arm (Studies 1 & 2) or the in-person PST arm (Study 2), this will occur for 3 days prior to, the day of, and 3 days after each scheduled PST session. For waitlist controls, this will occur for 7 days starting on Sunday of each assigned week.

**Appendix 3. Power analysis and statistical analysis plan – Aim 2 Pilot RCT**

1. **Power Analysis**

The primary objective of the Pilot RCT in the R61 phase is to evaluate neural target engagement based on the criteria (specified in B.1 below) in order to transition to the R33 phase. Research to examine neural targets as mechanisms of psychotherapy is an emerging field. No prespecified treatment difference of clinical importance or established effect sizes are available for our proposed neural target measures from fMRI. To address such early stage research, Julious(88) recommended that rather than powering in the traditional fashion to test a formal hypothesis of a (in truth unknown) desirable treatment difference, the sample size be selected in order to provide a given level of precision of the treatment effect estimates using the confidence interval (CI) approach, which Ma et al. have used in a completed NIH-funded pilot RCT.(89) Specifically, Julious(88) and others(90) recommended the use of precision intervals of prespecified standardized width to guide sample size determination. Julious(91) offered a further refinement to provide *Assurance* (akin to power) that a chosen standardized width of the precision interval contains the true mean treatment difference. To obtain a precision interval with a 2-sided standardized half-width of 0.5 (akin to a medium effect size) with 90% assurance, we have planned a sample size of 60 (n*_Trt_*=40, n*_Con_*=20), assuming ≥85% retention at 16 weeks. This will provide reliable estimates of *meaningful improvement* from week 0 to 16 in amygdala activity for nonconscious threat-related reactivity and in DLPFC activity for cognitive control among Lumen participants, compared with waitlist controls (target engagement criterion (1); see below). Tests of the 2 *a priori* neural targets—amygdala for emotional reactivity and DLPFC for cognitive control—are regarded as separate inferential domains, that is, not within one family of comparisons, and thus are not corrected for multiplicity (or familywise error rate) across domains.(92, 93) Importantly, as explained, the objective of the Pilot RCT is not to claim statistical significance of a prespecified key hypothesis but to obtain reliable effect estimates with precision for a decision on transition to the next phase. In this context, multiple testing is not required or recommended.(92, 93)

For criterion (2), we will examine correlations of change in amygdala activity with change in validated self-report measures of emotional reactivity (affect, worry), and correlations of change in DLPFC activity with change in validated self-report measures of cognitive control (problem solving, dysfunctional attitudes), among Lumen and waitlist participants. A sample size of 60 will be sufficient to detect a correlation coefficient of r=0.4 with 0.80 power and 2-sided α=0.05. We are not focused on smaller correlations (r<0.4) because their implications are likely to have more limited clinical relevance.

1. **Statistical Analysis Plan**

**B.1. Treatment effects on target engagement**

Based on literature and our preliminary data*,* we define neural targets *a priori* as activation of the amygdala for nonconscious threat-related emotion reactivity and activation of the DLPFC for cognitive control. The activation variables are from fMRI, which will be completed at weeks 0 and 16. Also, validated self-report surveys of emotional reactivity (worry, affect) and cognitive control (problem solving, dysfunctional attitudes) will be obtained at weeks 0 and 16. These self-report measures were chosen because of prior evidence for their role mediating the effect of PST on depressive symptoms.(94)

Note that improvement for emotional reactivity corresponds to a *decrease* in amygdala activity whereas improvement for cognitive control corresponds to an *increase* in DLPFC activity. For consistency in the presentation of analyses, the measure will be constructed such that higher positive values are better; thus, change toward a *higher value is improvement*.

Activation of either neural target satisfies 2 criteria:

1. Criterion 1. The level of task-evoked activation for either the amygdala for nonconscious threat-related emotional reactivity or for the DLPFC for cognitive control improves meaningfully from week 0 to 16 in the Lumen group compared with the waitlist control group.

The ENGAGE Phase 1 project (UIC IRB protocol #2015-1324) showed significant improvement in amygdala activation for nonconscious threat-related emotional reactivity from baseline to 2 months, and the standardized treatment effect was Cohen’s d=0.6. We also underscore that the 2-month time point in that project, which tested an integrated depression and obesity behavioral treatment, coincided with the start of weight loss treatment, which was sequenced after the first 5 PST sessions for depression, so measurements of neural targets at 2 months assessed the effect of PST alone. In ENGAGE, PST was delivered via in-person individual sessions with trained coaches.

The SPEAC project is at the frontier of research on developing a voice-based AI agent (Lumen) for PST and validating its effect on *a priori* neural targets, and the Pilot RCT (Study 1) is to estimate possible effects of target engagement, which, if promising, will support further investigation to confirm target engagement in Study 2. In this context, we apply a precision interval approach (see Section A above) to determine Criterion 1. We define that, compared with the waitlist control group, the Lumen PST treatment group will demonstrate a *meaningful improvement* in a neural target if the standardized between-group mean difference is at least Cohen’s d=0.3 in favor of Lumen (small effect; 50% of that in ENGAGE). At this effect size, the upper limit of the precision interval overlaps with d=0.8 (large effect) given a standardized half-width of 0.5 with 90% assurance that the interval contains the true mean difference based on the power analysis. We consider such a finding to be meaningful and will suffice Criterion 1.

1. Criterion 2. Change in the level of task-evoked activation of a neural target that meets Criterion 1 correlates with temporally concurrent change from week 0 to 16 in self-reported measures within the corresponding PST theory-based construct for emotional reactivity or cognitive control. As noted, we consider correlations of r=0.4 or greater meaningful. Specifically:
   1. If the amygdala target exhibits a meaningful improvement in the Lumen group vs. the waitlist control (Criterion 1), then we will examine whether that improvement is significantly correlated with improvements in validated self-report measures of emotional reactivity (worry, affect) over the same 0-to-16-week interval.
   2. If the DLPFC target exhibits a meaningful improvement in the Lumen group vs. the waitlist control (Criterion 1), then we will examine whether that improvement is significantly correlated with improvements in cognitive control (problem solving, dysfunctional attitudes) over the same 0-to-16-week interval.

Criterion 1 is tested via *t* test that compares the change from week 0 to 16 in the Lumen group with that change in the waitlist control group. Participants are analyzed based on the group to which they are assigned using all available data. The *t* test described above can be readily expanded for exploratory subgroup analyses by applying to each subgroup (e.g., female vs. male).

To test Criterion 2, we will examine the regression change of the amygdala activity from week 0 to 16 on change in self-reported measures of emotional reactivity (worry, affect) from week 0 to 16, controlling for baseline values of the self-reported measures and group assignment. We expect that the coefficient for the change in amygdala activation is significant and positive in the direction of improvement in its corresponding, validated emotional reactivity self-report measures and that this relationship may be significantly modified by group assignment. Similarly, we will examine the regression change of the DLPFC activity from week 0 to 16 on change in self-reported measures of cognitive control (problem solving, dysfunctional attitudes) from week 0 to 16, controlling for baseline values of the self-reported measures and group assignment. We expect that the coefficient for the change in DLPFC activation is significant and positive in the direction of improvement in its corresponding, cognitive control validated self-report measures and that this relationship may be significantly modified by group assignment.

***B.1.a. Supportive Voxelwise Whole Brain Analysis***

We will conduct an exploratory whole-brain analysis (1) to verify our *a priori* specification of neural targets, namely, amygdala for emotional reactivity and DLPFC for cognitive control, and (2) to identify any additional voxel clusters that are activated by our tasks and are associated with treatment response. This cluster-extent based thresholding analysis(95) will be carried out using the same SPM12 software used for neuroimage processing (see the *Study 1 Protocol Synopsis*, Forms E, which contains technical details). The identification of voxel clusters employs 2 criteria: (1) a primary threshold to identify activated voxels with a test level (e.g., P≤ 0.001), and (2) an extent threshold applied to clusters of contiguous voxels (neighbors at 5 of 6 voxel faces activated) based on an estimated distribution of cluster sizes, to control the family-wise error rate (FWER) for clusters (e.g., α_FWER≤ 0.05). Recently there has been concern that faulty statistical assumptions have led to high false-positive rates and over-large clusters that cross anatomical boundaries.(96-98) We follow the recommendations of Woo et al.(98) by setting P≤0.001 and employing nonparametric tests that avoid problematic Gaussian random field assumptions. This specification will provide sufficient sensitivity to allow detection of alternative anatomical clusters, which we can replicate using the same analysis strategy in Study 2.

**B.2. Treatment effects on PROs and ecological measures**

Secondary analyses will compare differences in discrete changes in PROs from week 0 to 16 and trajectories of change based on intensive time-series ecological measures between the Lumen and waitlist control groups. Analyses of the treatment effects on PROs will employ the same *t* test approach as described above for testing Criterion 1 for target engagement.

Longitudinal mixed modeling will be applied to analyses of the ecological measures (mood, stress, appraisal, and coping) where the design is Group$\times$Time=2$\times$8 as these measures will be obtained (using a validated measurement burst design)(99) daily for 7 days every 2 weeks (on weeks 0, 2, 4, 6, 8, 10, 12, 16). During active treatment in the Lumen group, this will occur for 3 days prior to, the day of, and 3 days after each scheduled PST session. For waitlist controls, this will occur for 7 days starting on Sunday of each assigned week. This allows us rich and detailed information on ambulatory changes over the course of treatment, as well as estimates of proximal responses to treatment within 3 days immediately post a PST session. For example, using the means of ecological measures over the 3 pre-PST days (to avoid confounding estimates with proximal treatment response) in the Lumen group and the first 3 days in the waitlist control group every 2 weeks as DVs, we will characterize their trajectory of change in terms of orthogonal linear, quadratic, and cubic components. We also will examine regression spline versions of these trajectories for visualization and interpretation. In a more exploratory vein, for each set of 7 daily ecological measures, collected biweekly, we will use piecewise linear models to characterize proximal responses to treatment based on the change of the 3 post-treatment days’ means from the 3 pre-treatment days’ means (omitting treatment day 4) in the Lumen group and the corresponding biweekly change of the last 3 days’ means from the first 3 days’ means (omitting day 4) in the waitlist control group. As these differences are nested within the 8 biweekly measurement bursts, and thus can be examined longitudinally, we can consider whether there is change over weeks in patients’ proximal responses to each PST session (in terms of the ecological measures). In all analyses, all available data for each DV will be used and missing data will be handled directly through maximum-likelihood estimation via mixed modeling, supplemented with imputation and sensitivity analysis (see Handling Missing Data in Section B.4 below).

**B.3. Dose effect analysis**

***B.3.a. Rationale for a standard dose***

Participants randomized to the Lumen treatment group will complete 8 scheduled sessions (4 weekly and then 4 biweekly) over 12 weeks using secure study iPads. The essential research question under study is whether the AI agent Lumen as a new form of PST delivery can meaningfully engage *a priori* neural targets as putative mechanisms of treatment for adults with moderate depressive and/or anxiety symptoms. Fairly extensive evidence has been gathered to establish the optimal dose for implementation of PST in general medical and community settings; this typically involves 6-8 sessions that are initially weekly and taper off to every 2-4 weeks over 12 weeks.(100, 101) Because this is a proof-of-concept project to develop an AI PST agent, we will design and train Lumen to emulate the standards developed for face-to-face delivery of one standardized, empirically supported dose. Dosing comparisons are thus made relative to appropriate implementation of PST and examination of differences in actual delivery (i.e., primarily number and length of sessions and, secondarily, indicators of intervention delivery fidelity).

***B.3.b. Definitions of primary and secondary dose measures***

Two primary dose measures will be the number of PST sessions and the length of PST sessions (in minutes) that participants complete with Lumen. Secondary dose measures will be each of the 2 primary measures weighted by the level of treatment fidelity. All Lumen-based PST sessions will be recorded and independently rated for fidelity by 2 PST master trainers using the validated 7-item PST Adherence and Competence Scale (PST-PAC).(64, 65)

In psychotherapy outcome research, dose is typically defined as the number of sessions of therapy as it is a natural quantitative unit of treatment applicable across types of psychotherapy.(102-105) In this study, we also include the length of sessions as a measure of dose because (1) it is possible that the number of sessions varies little among participants given the brevity and controlled delivery of the therapy and (2) session length is an important parameter for possible optimization of Lumen to improve participant engagement and efficacy. Note that these dose measures are defined at any stage of the treatment; for example, after 4 sessions have been offered, a participant might have attended 3 sessions (number) for a total of 118 minutes (length). In other words, the dose measures are time-related variables.

Similarly, therapeutic fidelity is another important parameter for optimization in Lumen. In our view, it is a proxy to the strength of medicine and its use in this context adjusts the implied assumption that one session (or the same length of a session) equals one unit or dose, except that the strength (potency) of active ingredients in psychotherapy cannot be measured or standardized in the same way as that of a medication. Although the level of fidelity may be defined differently (e.g., with varying weights of the item scores), the ratio of raw total score to the maximum is simple and easy to interpret and there is no empirical support, to our knowledge, for an alternative. Alternative definitions may be explored using data from this study, especially if they would provide a more nuanced understanding of the need for optimization of Lumen. We will construct fidelity-weighted versions of the dose measures, number and length, in order to adjust doses for conformance to standard PST. Assessing dose in the multiple ways as we propose will allow us to determine which measure is most accurate and to be employed in Study 2. Also, our methodology and results will make an important contribution as assessing dose effects of novel digital health interventions on mechanisms such as brain targets is complex and understudied.

***B.3.c. Dosing analyses***

Dosing analyses will be conducted **within the Lumen treatment group**. The primary dosing analysis will examine the relationship of neural target activation in the amygdala and DLPFC to the 4 measures of dose (number and length of PST sessions, fidelity-weighted number and length of PST sessions). The secondary dosing analysis will examine the relationship of PROs, ecological end-of-day assessments, and measures of treatment progress (e.g., PHQ9 and GAD7 across PST sessions) to the same 4 measures of dose. Note that given neural target activation measures and PROs are obtained at weeks 0 and 16, these analyses will focus on change over the full 16-week span of the study, whereas ecological end-of-day assessments and treatment progress measures are available for a total of 8 occasions each, and thus will be considered longitudinally.

***Neural targets***: The primary dosing analyses will consist in the separate regressions of change in activation from week 0 to 16 for the amygdala and DLPFC on each dose variable. This results in 8 separate regressions: amygdala on number of sessions, amygdala on length of sessions, amygdala on fidelity-weighted number, amygdala on fidelity-weighted length, and also DLPFC on the same 4 dose variables. Assumptions of linear regression will be verified and corrected through data transformation as needed. Our purpose is to identify variants of the dose variables with the strongest association (i.e., highest *R*^2^) with change over 16 weeks in the 2 neural targets. If the coefficient for dose in these regressions is positive and significant, it confirms that the dose variable is associated with higher change in neural target activation.

***Patient-reported outcomes***: Depression and anxiety symptoms, functioning, and health-related quality of life will be measured at week 0 and 16. Thus, the secondary dose analysis for PROs mirrors the primary analysis for neural targets. That is, change from week 0 to 16 in the PROs will be separately regressed on the 4 dose variants, resulting in separate PRO-dose measure regressions. As in the primary analysis, our purpose is to identify variants of the dose variables with the strongest association (i.e., highest *R*^2^) with change over 16 weeks in the PROs of interest.

***Ecological end-of-day assessments***: Ecological measures of mood, stress, appraisal, and coping will be available at weeks 0, 2, 4, 6, 8, 10, 12 and 16 (for a total of 8 occasions). Each week’s assessment is based on 7 contiguous days’ end-of-day assessments of the 4 ecological measures, with day 4 being the day of PST treatment in the Lumen group. Thus, the mean of the 3 pre-treatment days on these variables provides an indication of a participants’ status immediately prior to each PST session; the mean of the 3 post-treatment days on the same variables provides an indication of a participants’ status immediately post the session. For this secondary dosing analysis, we can mirror the dosing analysis by regressing change in the 7-day mean from week 0 to week 16 for each of the 4 ecological measures on the 4 dose variants. Importantly, there are 6 intermediate measurement occasions in addition to week 16, so we will be able to examine trajectories of change from week 0 to week 2, to week 4, and so on, to week 16. Moreover, for each ecological measure a set of time-related variables may be constructed, such as overall 7 days’ means, 3 pre-treatment days’ means, and differences between 3 pre-treatment days’ means and 3 post-treatment days’ means (omitting treatment day 4), so we will be able to examine trajectories of change in these variables. It is also important to note that the 4 dosing variables in these longitudinal regression models reflect the dose received up to that point in the treatment. This provides a view of how accurately the dosing variables perform for the ecological measures over the entire span of the treatment. These analyses will use time-varying mixed models where the DV (e.g., mood) and the dose measure vary over the 16-week period.(106-108) The models for each dose measure (e.g., number of sessions) will include separate polynomial terms for linear, quadratic, and cubic rates of change in the DV over time. Alternatively, we will model the log of the dose measure (e.g., number of sessions) considering that some studies of in-person psychotherapy reported a negatively accelerated relationship between dose and treatment progress, suggesting a pattern of successively diminishing returns with increasing dose.(102, 109, 110) We will compare model fit for the polynomial and logarithmic models using Schwartz Bayesian Information Criterion (BIC).

***Treatment progress measures***: To monitor treatment progress, symptoms (PHQ9, GAD7) and treatment acceptability (usability, user experience, and treatment alliance) will be measured in each PST session at weeks 1, 2, 3, 4, 6, 8, 10, and 12 (for a total of 8 occasions). Similar to the analyses of ecological measures as described above, we will implement polynomial and logarithmic models and compare model fit using BIC. These regressions’ $R^{2}$’s will allow us to examine in detail the relationship of treatment progress and cumulative dose, with fidelity-to-PST taken into account using the fidelity-weighted number and length of PST sessions. Additionally, as is common in psychotherapy in general, it is possible that not all participants will complete the full 8 sessions as some may drop out early owing to either lack of improvement or “enough” improvement based on self-assessment or yet other reasons related or unrelated to the treatment. Using the Good-Enough-Level (GEL) model, some studies of in-person psychotherapy showed that the rate of change varied as a function of treatment duration, namely, the rate of change (progress) was faster for patients attending fewer sessions but slower for those attending more sessions.(110-112) In other words, patients may remain in treatment until they improve. To explore whether a similar pattern is present for Lumen PST, we will apply the GEL modeling approach by extending the polynomial models for treatment progress measures, in particular, PHQ9 and GAD7, to include a fixed effect of total treatment dose (e.g., total number of sessions) and interactions between total treatment dose and linear, quadratic, and cubic rates of change. Essentially, the GEL model is a stratified analysis that examines dose effects across strata of participants with similar treatment dose (e.g., similar number of sessions attended), as opposed to aggregate dose effects across all participants.

Furthermore, we will conduct exploratory analyses of baseline characteristics in relation to the dose effects on neural targets, PROs, ecological measures, and treatment progress measures. We anticipate that baseline characteristics (e.g., severity at presentation, sex as a biological variable, sociodemographics) may be related to dosing estimates (e.g., via actual sessions completed). To explore these relationships, baseline characteristics and their interactions with dose measures will be added to the models as described above.

**B.4. Intention to Treat (ITT) and Handling Missing Data**

Clinical trials such as the one proposed must face the issues of dropout and missing data. We have put in place a strong plan to ensure maximum retention of participants through the course of the study, with a projected 85% or greater retention (see the *Recruitment and Retention Plan*). Data missing completely at random (MCAR) may be analyzed “as is” without imputation, as it does not introduce bias into estimates and inferences. More commonly, data are missing at random (MAR) owing to covariate-dependent events (e.g., irregular work shifts, children’s illness, transportation, and scheduling foul-ups). Our primary analysis technique, linear mixed models, is robust for MAR and provides unbiased estimates of model parameters without imputation. (One may impute as well, but the benefit is usually modestly improved power rather than changes to conclusions.) The more serious concern for behavioral interventions is the possibility that data will be missing not at random (MNAR). This occurs when dropout or missingness is related to the level of the outcome of interest (e.g., a participant’s level of depression or anxiety). If treatment is working well and symptoms of depression and anxiety have lessened, then participants may feel continuation in treatment is not necessary or beneficial. They drop out or miss sessions because the treatment seems to have worked. On the other hand, if participants experience no improvement in symptoms, they may drop out or miss sessions because they are discouraged that the treatment seems not to have worked. In either case, the loss of data is related to the outcomes under study and has the potential to bias the study’s conclusion about the intervention. Handling the problem of MNAR is difficult. First, there is no test for the presence of MNAR (*vs.* MAR). Second, there are many possible underlying sources of MNAR and there is no guarantee that explorations of missing data patterns will clearly reveal those sources. Third, missing data imputation for MNAR necessarily makes strong and untestable statistical assumptions, so that conclusions from imputed MNAR datasets must remain tentative. This picture reinforces our strong commitment to participant retention (see the Study 1 *Recruitment and Retention Plan*).

Missing data imputation implemented via SAS procedures MI, MIANALYZE, and MCMC will be carried out to reexamine all primary and secondary analysis.(113, 114) This effectively implements ITT as traditionally conducted. Specifically, both missing outcomes and covariates will be imputed under the assumption that data are MAR. The fully conditional specification (FCS) approach allows each variable type to be imputed with an appropriate model: linear, discriminant, logistic, or Poisson regression.(115-119) The algorithm for FCS is implemented in procedure MI: 100 imputed data sets will be created, each imputation is analyzed using MIXED, and finally MIANALYZE combines 100 sets into final estimates. This approach to MAR has been widely adopted given its robust performance.(120) Because linear mixed models estimated via direct likelihood as in MIXED offer unbiased estimates under MAR, the benefit of multiple imputation lies in compliance with the ITT fill-in requirement and modestly increased power. We anticipate these results to differ little from those provided by the primary analysis.

***B.4.a. Sensitivity of results to missing data assumptions***

These will be conducted by varying the models used for imputation.(121) There is no test for NMAR, but we will examine missing data frequency by condition and in covariate subgroups to detect and diagnose differential dropout. If warranted, we can shift to a Bayesian joint modeling approach to imputation using MCMC. The mathematical statistical underpinnings of sensitivity analysis for missing data imputation have advanced substantially (see, for example, the treatment of selection models in Chapter 8 of Daniels and Hogan(122)). However, the software implementation of these techniques has lagged behind the theory. Simpler approaches have been implemented in SAS at this time; however, the more complex techniques involving semiparametric specification of the Daniels and Hogan extrapolation model are not readily available. During the data collection years, this problem will likely be resolved. Here, we mention 3 approaches that seem feasible now: (1) With just a few data collection waves, the number of frequently occurring missing data patterns is likely 3 to 6 for neural targets and PROs and 8 to 10 for ecological assessments and treatment progress measures (some infrequent patterns can be grouped together); these patterns can be used to specify a pattern mixture model for the missing data. Such models merely include an indicator variable for each pattern and thereby provide a straightforward means to adjust results for missing data.(123) If results are not much altered by inclusion of pattern indicators, then concern about missing data assumptions is diminished; (2) Mallinckrodt et al.(124-126) reviewed simulation and other studies of the impact of MNAR on trial results (primary analyses like those we plan) and concluded that the impact of violations is rarely substantial. The R statistical environments add-on package ‘mice’ now has capabilities to create particular violations of MAR and MNAR assumptions, so that simulation-based exploration of violations using our own data will now be possible; and (3) SAS PROC MI has now incorporated an option to use a SCALE parameter (suggested range between 1.5 and 2.0) to explore the sensitivity of results to imputations relying on the MAR assumptions.

**Appendix 4. Study Event Windows**

**Event windows** (in Days)

| **Preferred windows (in Days)** | **ES** | **V1** | **EMA**#**1** | **R** |  |  |  |  |  |  |  |  |  |  |  |  |  |  | **V2** |
| --- | --- | --- | --- | --- | --- | --- | --- | --- | --- | --- | --- | --- | --- | --- | --- | --- | --- | --- | --- |
| Lumen PST: |  |  |  |  | **Orient.** | **S1** | **S2** | **S3** | **S4** |  | **S5** |  | **S6** |  | **S7** |  | **S8** |  |  |
| **Week (indexed from randomization) #:** from  to | -4  -2 | -2  -1 |  | 0 | 1  2 | 1  3 | 2  4 | 3  5 | 4  6 |  | 6  8 |  | 8  10 |  | 10  12 |  | 12  14 |  | **14**  **18** |
| IES-eligible to eConsent, max. days | 14 |  |  | **Randomization** |  |  |  |  |  |  |  |  |  |  |  |  |  |  |  |
| eConsent to V1, max. days |  | 14 |  |  |  |  |  |  |  |  |  |  |  |  |  |  |  |  |  |
| *IES-eligible to V1* *(rescreen if IES-V1>90d), max. days* | 28 | |  |  |  |  |  |  |  |  |  |  |  |  |  |  |  |  |  |
| V1 to EMA#1 start (automated), max. days |  | **1** |  |  |  |  |  |  |  |  |  |  |  |  |  |  |  |  |  |
| V1 to Randomization, max. days |  | **10** | |  |  |  |  |  |  |  |  |  |  |  |  |  |  |  |  |
| Randomization to Lumen Orientation Visit, max. days |  |  |  |  | 14 |  |  |  |  |  |  |  |  |  |  |  |  |  |  |
| Lumen Orientation to Session 1, max. days |  |  |  |  |  | 7 |  |  |  |  |  |  |  |  |  |  |  |  |  |
| Lumen Session to Session, max. days |  |  |  |  |  |  | 7 | 7 | 7 | 14 | | 14 | | 14 | | 14 | |  |  |
| Randomization to V2, target 112d (16 weeks) +/- allowable 14d window (14-18 weeks) |  |  |  |  |  |  |  |  |  |  |  |  |  |  |  |  |  |  | 112  +/-14 |

Abbreviations: IES, initial eligibility screening; EMA, ecological daily assessment; PST, problem solving treatment; S1-S8, sessions 1-8 with Lumen; V1, visit 1; V2, visit 2

# References

1. Kannampallil T, Ronneberg CR, Wittels NE, Kumar V, Lv N, Smyth JM, et al. Design and Formative Evaluation of a Virtual Voice-Based Coach for Problem-solving Treatment: Observational Study. JMIR Form Res. 2022;6(8):e38092. Epub 2022/08/16. doi: 10.2196/38092. PubMed PMID: 35969431; PubMed Central PMCID: PMCPMC9419044.

2. Tziortzi AC, Searle GE, Tzimopoulou S, Salinas C, Beaver JD, Jenkinson M, et al. Imaging dopamine receptors in humans with [11C]-(+)-PHNO: dissection of D3 signal and anatomy. Neuroimage. 2011;54(1):264-77. Epub 2010/07/06. doi: 10.1016/j.neuroimage.2010.06.044. PubMed PMID: 20600980.

3. Williams LM, Korgaonkar MS, Song YC, Paton R, Eagles S, Goldstein-Piekarski A, et al. Amygdala Reactivity to Emotional Faces in the Prediction of General and Medication-Specific Responses to Antidepressant Treatment in the Randomized iSPOT-D Trial. Neuropsychopharmacology. 2015;40(10):2398-408. Epub 2015/04/01. doi: 10.1038/npp.2015.89. PubMed PMID: 25824424; PubMed Central PMCID: PMCPMC4538354.

4. Goldstein-Piekarski AN, Korgaonkar MS, Green E, Suppes T, Schatzberg AF, Hastie T, et al. Human amygdala engagement moderated by early life stress exposure is a biobehavioral target for predicting recovery on antidepressants. Proc Natl Acad Sci U S A. 2016;113(42):11955-60. Epub 2016/10/30. doi: 10.1073/pnas.1606671113. PubMed PMID: 27791054; PubMed Central PMCID: PMCPMC5081583.

5. Ashburner J, Friston KJ. Unified segmentation. NeuroImage. 2005;26:839-51. doi: 10.1016/j.neuroimage.2005.02.018. PubMed PMID: 15955494.

6. Williams LM. Defining biotypes for depression and anxiety based on large-scale circuit dysfunction: a theoretical review of the evidence and future directions for clinical translation. Depress Anxiety. 2017;34(1):9-24. Epub 2016/10/21. doi: 10.1002/da.22556. PubMed PMID: 27653321; PubMed Central PMCID: PMCPMC5702265.

7. Williams LM, Pines A, Goldstein-Piekarski AN, Rosas LG, Kullar M, Sacchet MD, et al. The ENGAGE study: Integrating neuroimaging, virtual reality and smartphone sensing to understand self-regulation for managing depression and obesity in a precision medicine model. Behav Res Ther. 2018;101:58-70. Epub 2017/10/28. doi: 10.1016/j.brat.2017.09.012. PubMed PMID: 29074231; PubMed Central PMCID: PMCPMC8109191.

8. Lv N, Ajilore OA, Ronneberg CR, Venditti EM, Snowden MB, Lavori PW, et al. The ENGAGE-2 study: Engaging self-regulation targets to understand the mechanisms of behavior change and improve mood and weight outcomes in a randomized controlled trial (Phase 2). Contemp Clin Trials. 2020;95:106072. Epub 2020/07/06. doi: 10.1016/j.cct.2020.106072. PubMed PMID: 32621905; PubMed Central PMCID: PMCPMC8136578.

9. Goldstein-Piekarski AN, Ball TM, Samara Z, Staveland BR, Keller AS, Fleming SL, et al. Mapping Neural Circuit Biotypes to Symptoms and Behavioral Dimensions of Depression and Anxiety. Biol Psychiatry. 2022;91(6):561-71. Epub 2021/09/07. doi: 10.1016/j.biopsych.2021.06.024. PubMed PMID: 34482948.

10. Williams LM. Precision psychiatry: a neural circuit taxonomy for depression and anxiety. Lancet Psychiatry. 2016;3(5):472-80. Epub 2016/05/08. doi: 10.1016/S2215-0366(15)00579-9. PubMed PMID: 27150382; PubMed Central PMCID: PMCPMC4922884.

11. Yarkoni T, Poldrack R, Nichols T, Van Essen D, Wager T. NeuroSynth: a new platform for large-scale automated synthesis of human functional neuroimaging data2011.

12. Tzourio-Mazoyer N, Landeau B, Papathanassiou D, Crivello F, Etard O, Delcroix N, et al. Automated anatomical labeling of activations in SPM using a macroscopic anatomical parcellation of the MNI MRI single-subject brain. Neuroimage. 2002;15(1):273-89. Epub 2002/01/05. doi: 10.1006/nimg.2001.0978. PubMed PMID: 11771995.

13. Roehrig C. Mental disorders top the list of the most costly conditions in the United States: $201 billion. Health affairs (Project Hope). 2016;35(6):1130-5. Epub 2016/05/20. doi: 10.1377/hlthaff.2015.1659. PubMed PMID: 27193027; PubMed Central PMCID: PMCPMCID:NA.

14. Greenberg PE, Fournier AA, Sisitsky T, Pike CT, Kessler RC. The economic burden of adults with major depressive disorder in the United States (2005 and 2010). The Journal of clinical psychiatry. 2015;76(2):155-62. Epub 2015/03/06. doi: 10.4088/JCP.14m09298. PubMed PMID: 25742202; PubMed Central PMCID: PMCPMCID:NA.

15. National Institute of Mental Health. Major Depression Among Adults 2015 [cited 2017 October 11]. Available from: <https://www.nimh.nih.gov/health/statistics/prevalence/major-depression-among-adults.shtml>.

16. National Institute of Mental Health. Any anxiety disorder among adults 2015 [cited 2017 November 21]. Available from: <https://www.nimh.nih.gov/health/statistics/prevalence/any-anxiety-disorder-among-adults.shtml>.

17. Anxiety and Depression Association of America. Facts & statistics 2017 [cited 2017 November 21]. Available from: <https://adaa.org/about-adaa/press-room/facts-statistics>.

18. Rodriguez MR, Nuevo R, Chatterji S, Ayuso-Mateos JL. Definitions and factors associated with subthreshold depressive conditions: a systematic review. BMC Psychiatry. 2012;12:181. Epub 2012/11/01. doi: 10.1186/1471-244X-12-181. PubMed PMID: 23110575; PubMed Central PMCID: PMCPMC3539957.

19. Meeks TW, Vahia IV, Lavretsky H, Kulkarni G, Jeste DV. A tune in "a minor" can "b major": a review of epidemiology, illness course, and public health implications of subthreshold depression in older adults. Journal of affective disorders. 2011;129(1-3):126-42. Epub 2010/10/12. doi: 10.1016/j.jad.2010.09.015. PubMed PMID: 20926139; PubMed Central PMCID: PMCPMC3036776.

20. Haller H, Cramer H, Lauche R, Gass F, Dobos GJ. The prevalence and burden of subthreshold generalized anxiety disorder: a systematic review. BMC Psychiatry. 2014;14(1):128. doi: 10.1186/1471-244X-14-128. PubMed PMID: 24886240; PubMed Central PMCID: PMCPMC4048364.

21. Gonzalez HM, Vega WA, Williams DR, Tarraf W, West BT, Neighbors HW. Depression care in the United States: too little for too few. Arch Gen Psychiatry. 2010;67(1):37-46. Epub 2010/01/06. doi: 10.1001/archgenpsychiatry.2009.168. PubMed PMID: 20048221; PubMed Central PMCID: PMCPMC2887749.

22. Kessler RC, Berglund P, Demler O, Jin R, Koretz D, Merikangas KR, et al. The epidemiology of major depressive disorder: results from the National Comorbidity Survey Replication (NCS-R). Jama. 2003;289(23):3095-105. Epub 2003/06/19. doi: 10.1001/jama.289.23.3095. PubMed PMID: 12813115; PubMed Central PMCID: PMCPMCID:NA.

23. Cook BL, Trinh NH, Li Z, Hou SS, Progovac AM. Trends in racial-ethnic disparities in access to mental health care, 2004-2012. Psychiatric services (Washington, DC). 2017;68(1):9-16. Epub 2016/08/02. doi: 10.1176/appi.ps.201500453. PubMed PMID: 27476805; PubMed Central PMCID: PMCPMC5895177.

24. van Schaik DJ, Klijn AF, van Hout HP, van Marwijk HW, Beekman AT, de Haan M, et al. Patients' preferences in the treatment of depressive disorder in primary care. General hospital psychiatry. 2004;26(3):184-9. Epub 2004/05/04. doi: 10.1016/j.genhosppsych.2003.12.001. PubMed PMID: 15121346; PubMed Central PMCID: PMCPMCID:NA.

25. Regier DA, Narrow WE, Rae DS, Manderscheid RW, Locke BZ, Goodwin FK. The de facto US mental and addictive disorders service system. Epidemiologic catchment area prospective 1-year prevalence rates of disorders and services. Arch Gen Psychiatry. 1993;50(2):85-94. Epub 1993/02/01. doi: 10.1001/archpsyc.1993.01820140007001. PubMed PMID: 8427558; PubMed Central PMCID: PMCPMCID:NA.

26. McHugh RK, Whitton SW, Peckham AD, Welge JA, Otto MW. Patient preference for psychological vs pharmacologic treatment of psychiatric disorders: a meta-analytic review. The Journal of clinical psychiatry. 2013;74(6):595-602. Epub 2013/07/12. doi: 10.4088/JCP.12r07757. PubMed PMID: 23842011; PubMed Central PMCID: PMCPMC4156137.

27. Hirschfeld RM, Keller MB, Panico S, Arons BS, Barlow D, Davidoff F, et al. The National Depressive and Manic-Depressive Association consensus statement on the undertreatment of depression. Jama. 1997;277(4):333-40. Epub 1997/01/22. PubMed PMID: 9002497; PubMed Central PMCID: PMCPMCID:NA.

28. Fortney JC, Harman JS, Xu S, Dong F. The association between rural residence and the use, type, and quality of depression care. The Journal of rural health : official journal of the American Rural Health Association and the National Rural Health Care Association. 2010;26(3):205-13. Epub 2010/07/17. doi: 10.1111/j.1748-0361.2010.00290.x. PubMed PMID: 20633088; PubMed Central PMCID: PMCPMCID:NA.

29. Sorkin DH, Murphy M, Nguyen H, Biegler KA. Barriers to Mental Health Care for an Ethnically and Racially Diverse Sample of Older Adults. Journal of the American Geriatrics Society. 2016;64(10):2138-43. Epub 2016/10/21. doi: 10.1111/jgs.14420. PubMed PMID: 27565017; PubMed Central PMCID: PMCPMC5937991.

30. Leong FT, Kalibatseva Z. Cross-cultural barriers to mental health services in the United States. Cerebrum : the Dana forum on brain science. 2011;2011:5. Epub 2011/03/01. PubMed PMID: 23447774; PubMed Central PMCID: PMCPMC3574791.

31. Proudfoot J, Parker G, Hadzi Pavlovic D, Manicavasagar V, Adler E, Whitton A. Community attitudes to the appropriation of mobile phones for monitoring and managing depression, anxiety, and stress. Journal of Medical Internet Research. 2010;12(5):e64. Epub 19.12.2010. doi: 10.2196/jmir.1475. PubMed PMID: 21169174; PubMed Central PMCID: PMCPMC3057321.

32. DeAndrea DC. Testing the proclaimed affordances of online support groups in a nationally representative sample of adults seeking mental health assistance. Journal of Health Communication. 2015;20(2):147-56. doi: 10.1080/10810730.2014.914606. PubMed PMID: 25116383; PubMed Central PMCID: PMCPMCID:NA.

33. Cuijpers P, Marks IM, van Straten A, Cavanagh K, Gega L, Andersson G. Computer-aided psychotherapy for anxiety disorders: a meta-analytic review. Cognitive behaviour therapy. 2009;38(2):66-82. Epub 2010/02/26. doi: 10.1080/16506070802694776. PubMed PMID: 20183688; PubMed Central PMCID: PMCPMCID:NA.

34. Andrews G, Cuijpers P, Craske MG, McEvoy P, Titov N. Computer therapy for the anxiety and depressive disorders is effective, acceptable and practical health care: a meta-analysis. PloS one. 2010;5(10):e13196. Epub 2010/10/23. doi: 10.1371/journal.pone.0013196. PubMed PMID: 20967242; PubMed Central PMCID: PMCPMC2954140.

35. National Institute for Health and Care Excellence. Computerised cognitive behaviour therapy for depression and anxiety 2013 [cited 2018 April 8]. Available from: <https://www.nice.org.uk/guidance/ta97/chapter/1-Guidance>.

36. Firth J, Torous J, Nicholas J, Carney R, Pratap A, Rosenbaum S, et al. The efficacy of smartphone‐based mental health interventions for depressive symptoms: a meta‐analysis of randomized controlled trials. World Psychiatry. 2017;16(3):287-98. doi: 10.1002/wps.20472. PubMed PMID: 28941113; PubMed Central PMCID: PMCPMC5608852.

37. Firth J, Torous J, Nicholas J, Carney R, Rosenbaum S, Sarris J. Can smartphone mental health interventions reduce symptoms of anxiety? A meta-analysis of randomized controlled trials. Journal of affective disorders. 2017;218:15-22. Epub 2017/04/30. doi: 10.1016/j.jad.2017.04.046. PubMed PMID: 28456072; PubMed Central PMCID: PMCPMCID:NA.

38. Bohn D. Amazon says 100 million Alexa devices have been sold — what’s next? The Verge, 2019.

39. Kinsella B. RBC Analyst Says 52 Million Google Home Devices Sold to Date and Generating $3.4 Billion in 2018 Revenue 2018. Available from: <https://voicebot.ai/2018/12/24/rbc-analyst-says-52-million-google-home-devices-sold-to-date-and-generating-3-4-billion-in-2018-revenue/>.

40. Topol EJ. High-performance medicine: the convergence of human and artificial intelligence. Nature Medicine. 2019;25(1):44-56. doi: 10.1038/s41591-018-0300-7. PubMed PMID: 30617339; PubMed Central PMCID: PMCPMCID:NA.

41. Topol E. Deep Medicine: How Artificial Intelligence Can Make Healthcare Human Again. First ed: Basic Books; 2019. 400 p.

42. Lucas GM, Gratch J, King A, Morency L-P. It’s only a computer: Virtual humans increase willingness to disclose. Computers in Human Behavior. 2014;37:94-100. doi: <https://doi.org/10.1016/j.chb.2014.04.043>. PubMed PMID: :NA; PubMed Central PMCID: PMCPMCID:NA.

43. Nass C, Moon Y. Machines and mindlessness: social responses to computers. Journal of Social Issues. 2000;56:81-103. doi: 10.1111/0022-4537.00153. PubMed PMID: :NA; PubMed Central PMCID: PMCPMCID:NA.

44. Nass C, Steuer J, Tauber ER. Computers are social actors. Proceedings of the SIGCHI Conference on Human Factors in Computing Systems; Boston, Massachusetts, USA. 191703: ACM; 1994. p. 72-8.

45. Ho A, Hancock J, Miner AS. Psychological, relational, and emotional effects of self-disclosure after conversations with a chatbot. The Journal of communication. 2018;68(4):712-33. Epub 2018/08/14. doi: 10.1093/joc/jqy026. PubMed PMID: 30100620; PubMed Central PMCID: PMCPMC6074615.

46. Bickmore T, Gruber A, Picard R. Establishing the computer-patient working alliance in automated health behavior change interventions. Patient education and counseling. 2005;59(1):21-30. Epub 2005/10/04. doi: 10.1016/j.pec.2004.09.008. PubMed PMID: 16198215; PubMed Central PMCID: PMCPMCID:NA.

47. Bickmore TW, Caruso L, Clough-Gorr K, Heeren T. ‘It’s just like you talk to a friend’ relational agents for older adults. Interacting with Computers. 2005;17(6):711–35. doi: <https://doi.org/10.1016/j.intcom.2005.09.002>. PubMed PMID: :NA; PubMed Central PMCID: PMCPMCID:NA.

48. Bickmore TW, Pfeifer LM, Byron D, Forsythe S, Henault LE, Jack BW, et al. Usability of conversational agents by patients with inadequate health literacy: evidence from two clinical trials. Journal of Health Communication. 2010;15 Suppl 2:197-210. Epub 2010/09/29. doi: 10.1080/10810730.2010.499991. PubMed PMID: 20845204; PubMed Central PMCID: PMCPMCID:NA.

49. Laranjo L, Dunn AG, Tong HL, Kocaballi AB, Chen J, Bashir R, et al. Conversational agents in healthcare: a systematic review. Journal of the American Medical Informatics Association : JAMIA. 2018;25(9):1248-58. Epub 2018/07/17. doi: 10.1093/jamia/ocy072. PubMed PMID: 30010941; PubMed Central PMCID: PMCPMC6118869.

50. Ring L, Bickmore T, Pedrelli P. An affectively aware virtual therapist for depression counseling 2017 [cited 2017 October 17]. Available from: <http://mentalhealth.media.mit.edu/wp-content/uploads/sites/46/2016/04/CHI2016-MentalHealth-lring.pdf>.

51. Bickmore T, Gruber A. Relational agents in clinical psychiatry. Harvard review of psychiatry. 2010;18(2):119-30. Epub 2010/03/20. doi: 10.3109/10673221003707538. PubMed PMID: 20235777; PubMed Central PMCID: PMCPMCID:NA.

52. Fitzpatrick KK, Darcy A, Vierhile M. Delivering cognitive behavior therapy to young adults with symptoms of depression and anxiety using a fully automated conversational agent (Woebot): a randomized controlled trial. JMIR mental health. 2017;4(2):e19. Epub 2017/06/08. doi: 10.2196/mental.7785. PubMed PMID: 28588005; PubMed Central PMCID: PMCPMC5478797.

53. Pereira J, Diaz O. Using health chatbots for behavior change: a mapping study. Journal of medical systems. 2019;43(5):135. Epub 2019/04/06. doi: 10.1007/s10916-019-1237-1. PubMed PMID: 30949846; PubMed Central PMCID: PMCPMCID:NA.

54. National Institute of Mental Health. Strategic research priorities overview 2019 [cited 2019 September 6]. Available from: <https://www.nimh.nih.gov/about/strategic-planning-reports/strategic-research-priorities/index.shtml>.

55. National Institute of Mental Health. Opportunities and challenges of developing information technologies on behavioral and social science clinical research Year N/A [cited 2019 September 6]. Available from: <https://www.nimh.nih.gov/about/advisory-boards-and-groups/namhc/reports/opportunities-and-challenges-of-developing-information-technologies-on-behavioral-and-social-science-clinical-research.shtml>.

56. Hart SG, Stavenland LE. Development of NASA-TLX (Task Load Index): Results of empirical and theoretical research. In: Hancock P, Meshkati N, editors. Human Mental Workload. Amsterdam, The Netherlands: Elsevier; 1988.

57. Schrepp M, Hinderks A, Thomaschewski Jr. Design and evaluation of a short version of the User Experience Questionnaire (UEQ-S). Int J Interact Multimed Artif Intell. 2017;4(Regular Issue):103-8. doi: 10.9781/ijimai.2017.09.001. PubMed PMID: :NA; PubMed Central PMCID: PMCPMCID:NA.

58. Hatcher RL, Gillaspy JA. Development and validation of a revised short version of the working alliance inventory. Psychotherapy Research. 2006;16(1):12-25. doi: 10.1080/10503300500352500. PubMed PMID: :NA; PubMed Central PMCID: PMCPMCID:NA.

59. Munder T, Wilmers F, Leonhart R, Linster Hans W, Barth J. Working Alliance Inventory‐Short Revised (WAI‐SR): psychometric properties in outpatients and inpatients. Clinical Psychology & Psychotherapy. 2009;17(3):231-9. doi: 10.1002/cpp.658. PubMed PMID: 20013760; PubMed Central PMCID: PMCPMCID:NA.

60. Kiluk BD, Serafini K, Frankforter T, Nich C, Carroll KM. Only Connect: the working alliance in computer-based cognitive behavioral therapy. Behaviour research and therapy. 2014;63:139-46. doi: 10.1016/j.brat.2014.10.003. PubMed PMID: 25461789; PubMed Central PMCID: PMCPMC4408209.

61. Bickmore TW, Mitchell SE, Jack BW, Paasche-Orlow MK, Pfeifer LM, Odonnell J. Response to a relational agent by hospital patients with depressive symptoms. Interacting with computers. 2010;22(4):289-98. doi: 10.1016/j.intcom.2009.12.001. PubMed PMID: 20628581; PubMed Central PMCID: PMCPMC2901553.

62. van der Vaart R, Drossaert C. Development of the Digital Health Literacy instrument: measuring a broad spectrum of Health 1.0 and Health 2.0 Skills. Journal of Medical Internet Research. 2017;19(1):e27. doi: 10.2196/jmir.6709. PubMed PMID: 28119275; PubMed Central PMCID: PMCPMC5358017.

63. Polson PG, Lewis C, Rieman J, Wharton C. Cognitive walkthroughs: a method for theory-based evaluation of user interfaces. International Journal of Man-Machine Studies. 1992;36(5):741-73. doi: <https://doi.org/10.1016/0020-7373(92)90039-N>. PubMed PMID: :NA; PubMed Central PMCID: PMCPMCID:NA.

64. Oxman TE, Hegel MT, Hull JG, Dietrich AJ. Problem-solving treatment and coping styles in primary care for minor depression. Journal of consulting and clinical psychology. 2008;76(6):933-43. Epub 2008/12/03. doi: 10.1037/a0012617. PubMed PMID: 19045962; PubMed Central PMCID: PMCPMC2593861.

65. Hegel MT, Dietrich AJ, Seville JL, Jordan CB. Training residents in problem-solving treatment of depression: a pilot feasibility and impact study. Family medicine. 2004;36(3):204-8. Epub 2004/03/05. PubMed PMID: 14999578; PubMed Central PMCID: PMCPMCID:NA.

66. New C, Xiao L, Ma J. Acculturation and overweight-related attitudes and behavior among obese Hispanic adults in the United States. Obesity (Silver Spring, Md). 2013;21(11):2396-404. Epub 2013/05/21. doi: 10.1002/oby.20146. PubMed PMID: 23687100.

67. Pocock SJ, Simon R. Sequential treatment assignment with balancing for prognostic factors in the controlled clinical trial. Biometrics. 1975;31(1):103-15. Epub 1975/03/01. PubMed PMID: 1100130; PubMed Central PMCID: PMCPMCID:NA.

68. Therneau TM. How many stratification factors are "too many" to use in a randomization plan? Controlled clinical trials. 1993;14(2):98-108. Epub 1993/04/01. PubMed PMID: 8500309; PubMed Central PMCID: PMCPMCID:NA.

69. Efron B. Forcing a sequential experiment to be balanced. Biometrika. 1971;58(3):403-17. doi: 10.2307/2334377. PubMed PMID: :NA; PubMed Central PMCID: PMCPMCID:NA.

70. Kuznetsova OM, Tymofyeyev Y. Preserving the allocation ratio at every allocation with biased coin randomization and minimization in studies with unequal allocation. Statistics in medicine. 2012;31(8):701-23. Epub 2011/12/14. doi: 10.1002/sim.4447. PubMed PMID: 22161821; PubMed Central PMCID: PMCPMCID:NA.

71. Lv N, Ajilore OA, Ronneberg CR, Venditti EM, Snowden MB, Lavori PW, et al. The ENGAGE-2 study: Engaging self-regulation targets to understand the mechanisms of behavior change and improve mood and weight outcomes in a randomized controlled trial (Phase 2). Contemp Clin Trials. 2020:106072. Epub 2020/07/06. doi: 10.1016/j.cct.2020.106072. PubMed PMID: 32621905.

72. Hsieh H-F, Shannon SE. Three approaches to qualitative content analysis. Qualitative Health Research. 2005;15(9):1277-88. doi: 10.1177/1049732305276687. PubMed PMID: 16204405; PubMed Central PMCID: PMCPMCID:NA.

73. Meyer TJ, Miller ML, Metzger RL, Borkovec TD. Development and validation of the Penn State Worry Questionnaire. Behav Res Ther. 1990;28(6):487-95. Epub 1990/01/01. doi: 10.1016/0005-7967(90)90135-6. PubMed PMID: 2076086; PubMed Central PMCID: PMCPMCID:NA.

74. Watson D, Clark LA, Tellegen A. Development and validation of brief measures of positive and negative affect: The PANAS scales. Journal of Personality and Social Psychology. 1988;54(6):1063-70. doi: 10.1037/0022-3514.54.6.1063. PubMed PMID: :NA; PubMed Central PMCID: PMCPMCID:NA.

75. D’Zurilla T, Nezu A, Maydeu-Olivares A. Manual for the Social Problem-Solving Inventory-Revised. North Tonawanda, NY: Multi-Health Systems; 2002.

76. Weissman AN. The Dysfunctional Attitude Scale: A Validation Study. Publicly Accessible Penn Dissertations. 1182.: University of Pennsylvania; 1979.

77. Snaith R, Zigmond A. The Hospital Anxiety and Depression Scale manual. Windsor, Berkshire (UK): Nfer-Nelson; 1994.

78. Zigmond AS, Snaith RP. The hospital anxiety and depression scale. Acta psychiatrica Scandinavica. 1983;67(6):361-70. Epub 1983/06/01. PubMed PMID: 6880820; PubMed Central PMCID: PMCPMCID:NA.

79. Sheehan KH, Sheehan DV. Assessing treatment effects in clinical trials with the discan metric of the Sheehan Disability Scale. International clinical psychopharmacology. 2008;23(2):70-83. Epub 2008/02/28. doi: 10.1097/YIC.0b013e3282f2b4d6. PubMed PMID: 18301121; PubMed Central PMCID: PMCPMCID:NA.

80. Kessler RC, Barber C, Beck A, Berglund P, Cleary PD, McKenas D, et al. The World Health Organization Health and Work Performance Questionnaire (HPQ). Journal of occupational and environmental medicine. 2003;45(2):156-74. Epub 2003/03/11. PubMed PMID: 12625231; PubMed Central PMCID: PMCPMCID:NA.

81. Kessler RC, Ames M, Hymel PA, Loeppke R, McKenas DK, Richling DE, et al. Using the World Health Organization Health and Work Performance Questionnaire (HPQ) to evaluate the indirect workplace costs of illness. Journal of occupational and environmental medicine. 2004;46(6 Suppl):S23-37. Epub 2004/06/15. PubMed PMID: 15194893; PubMed Central PMCID: PMCPMCID:NA.

82. Ware J, Jr., Kosinski M, Keller SD. A 12-Item Short-Form Health Survey: construction of scales and preliminary tests of reliability and validity. Medical care. 1996;34(3):220-33. Epub 1996/03/01. PubMed PMID: 8628042; PubMed Central PMCID: PMCPMCID:NA.

83. Grasso DJ, Briggs-Gowan MJ, Ford JD, Carter AS. The Epidemic – Pandemic Impacts Inventory (EPII) 2020 [cited 2021 March 24]. Available from: <https://health.uconn.edu/psychiatry/wp-content/uploads/sites/51/2020/05/EPII-Main-V1.pdf>.

84. Koenig HG, Büssing A. The Duke University Religion Index (DUREL): A Five-Item Measure for Use in Epidemological Studies. Religions. 2010;1(1):78-85.

85. Berkman LF, Syme SL. Social networks, host resistance, and mortality: a nine-year follow-up study of Alameda County residents. Am J Epidemiol. 1979;109(2):186-204. Epub 1979/02/01. doi: 10.1093/oxfordjournals.aje.a112674. PubMed PMID: 425958.

86. Ford ES, Loucks EB, Berkman LF. Social integration and concentrations of C-reactive protein among US adults. Ann Epidemiol. 2006;16(2):78-84. Epub 2005/11/08. doi: 10.1016/j.annepidem.2005.08.005. PubMed PMID: 16271297.

87. Hughes ME, Waite LJ, Hawkley LC, Cacioppo JT. A Short Scale for Measuring Loneliness in Large Surveys: Results From Two Population-Based Studies. Res Aging. 2004;26(6):655-72. Epub 2008/05/28. doi: 10.1177/0164027504268574. PubMed PMID: 18504506; PubMed Central PMCID: PMCPMC2394670.

88. Julious SA, Patterson SD. Sample sizes for estimation in clinical research. Pharmaceutical Statistics. 2004;3(3):213-5. doi: 10.1002/pst.125. PubMed PMID: :NA; PubMed Central PMCID: PMCPMCID:NA.

89. Ma J, Strub P, Lv N, Xiao L, Camargo CA, Jr., Buist AS, et al. Pilot randomised trial of a healthy eating behavioural intervention in uncontrolled asthma. The European Respiratory Journal. 2016;47(1):122-32. Epub 2015/10/24. doi: 10.1183/13993003.00591-2015. PubMed PMID: 26493792; PubMed Central PMCID: PMCPMC5136475.

90. Sheiner LB. Learning versus confirming in clinical drug development. Clinical pharmacology and therapeutics. 1997;61(3):275-91. Epub 1997/03/01. doi: 10.1016/s0009-9236(97)90160-0. PubMed PMID: 9084453; PubMed Central PMCID: PMCPMCID:NA.

91. Julious SA. Sample sizes for clinical trials with Normal data. Statistics in medicine. 2004;23(12):1921-86. doi: 10.1002/sim.1783. PubMed PMID: :NA; PubMed Central PMCID: PMCPMCID:NA.

92. Feise RJ. Do multiple outcome measures require p-value adjustment? BMC medical research methodology. 2002;2:8. Epub 2002/06/19. PubMed PMID: 12069695; PubMed Central PMCID: PMCPMC117123.

93. Althouse AD. Adjust for multiple comparisons? It's not that simple. The Annals of thoracic surgery. 2016;101(5):1644-5. Epub 2016/04/24. doi: 10.1016/j.athoracsur.2015.11.024. PubMed PMID: 27106412; PubMed Central PMCID: PMCPMCID:NA.

94. Warmerdam L, van Straten A, Jongsma J, Twisk J, Cuijpers P. Online cognitive behavioral therapy and problem-solving therapy for depressive symptoms: Exploring mechanisms of change. Journal of behavior therapy and experimental psychiatry. 2010;41(1):64-70. Epub 2009/11/17. doi: 10.1016/j.jbtep.2009.10.003. PubMed PMID: 19913781; PubMed Central PMCID: PMCPMCID;NA.

95. Forman SD, Cohen JD, Fitzgerald M, Eddy WF, Mintun MA, Noll DC. Improved assessment of significant activation in functional magnetic resonance imaging (fMRI): use of a cluster-size threshold. Magnetic resonance in medicine. 1995;33(5):636-47. Epub 1995/05/01. doi: 10.1002/mrm.1910330508. PubMed PMID: 7596267; PubMed Central PMCID: PMCPMCID:NA.

96. Carter CS, Lesh TA, Barch DM. Thresholds, power, and sample sizes in clinical neuroimaging. Biological Psychiatry: Cognitive Neuroscience and Neuroimaging. 2016;1(2):99-100. doi: 10.1016/j.bpsc.2016.01.005. PubMed PMID: :NA; PubMed Central PMCID: PMCPMCID:NA.

97. Eklund A, Nichols TE, Knutsson H. Cluster failure: Why fMRI inferences for spatial extent have inflated false-positive rates. Proc Natl Acad Sci U S A. 2016;113(28):7900-5. Epub 2016/07/01. doi: 10.1073/pnas.1602413113. PubMed PMID: 27357684; PubMed Central PMCID: PMCPMC4948312.

98. Woo CW, Krishnan A, Wager TD. Cluster-extent based thresholding in fMRI analyses: pitfalls and recommendations. Neuroimage. 2014;91:412-9. Epub 2014/01/15. doi: 10.1016/j.neuroimage.2013.12.058. PubMed PMID: 24412399; PubMed Central PMCID: PMCPMC4214144.

99. Sliwinski MJ. Measurement-burst designs for social health research. Social and Personality Psychology Compass. 2008;2(1):245-61. doi: 10.1111/j.1751-9004.2007.00043.x. PubMed PMID: :NA; PubMed Central PMCID: PMCPMCID:NA.

100. Hegel M, Barrett J, Oxman T, Mynors-Wallis L, Gath D. Problem-Solving Treatment for Primary Care (PST-PC): A Treatment Manual for Depression. Hanover, New Hampshire: Dartmouth University; 1999.

101. Cape J, Whittington C, Buszewicz M, Wallace P, Underwood L. Brief psychological therapies for anxiety and depression in primary care: meta-analysis and meta-regression. BMC medicine. 2010;8:38. Epub 2010/06/29. doi: 10.1186/1741-7015-8-38. PubMed PMID: 20579335; PubMed Central PMCID: PMCPMC2908553.

102. Howard KI, Kopta SM, Krause MS, Orlinsky DE. The dose-effect relationship in psychotherapy. The American psychologist. 1986;41(2):159-64. Epub 1986/02/01. PubMed PMID: 3516036; PubMed Central PMCID: PMCPMCID:NA.

103. Hansen NB, Lambert MJ, Forman EM. The psychotherapy dose-response effect and its implications for treatment delivery services. Clin Psychol Sci Prac. 2002;9(3):329-43. doi: 10.1093/clipsy.9.3.329. PubMed PMID: :NA; PubMed Central PMCID: PMCPMCID:NA.

104. Ginestet CE, Emsley R, Landau S. Dose-response modeling in mental health using stein-like estimators with instrumental variables. Statistics in medicine. 2017;36(11):1696-714. Epub 2017/02/22. doi: 10.1002/sim.7265. PubMed PMID: 28222485; PubMed Central PMCID: PMCPMC5434902.

105. Robinson L, Delgadillo J, Kellett S. The dose-response effect in routinely delivered psychological therapies: A systematic review. Psychotherapy research : journal of the Society for Psychotherapy Research. 2019:1-18. Epub 2019/01/22. doi: 10.1080/10503307.2019.1566676. PubMed PMID: 30661486; PubMed Central PMCID: PMCPMCID:NA.

106. Diggle P, Heagerty, P., Liang, K.-Y. and Zeger, S. Analysis of Longitudinal Data. 2nd ed. Oxford, UK: Oxford University Press; 2002.

107. Fitzmaurice GM, Laird, N.M. and Ware, J.H. Applied longitudinal analysis. 2nd ed. Hoboken, NJ: John Wiley & Sons, Inc; 2011.

108. Hedeker D, Gibbons R. Longitudinal data analysis. Hoboken, NJ: Wiley-Interscience; 2006.

109. Baldwin SA, Berkeljon A, Atkins DC, Olsen JA, Nielsen SL. Rates of change in naturalistic psychotherapy: contrasting dose-effect and good-enough level models of change. J Consult Clin Psychol. 2009;77(2):203-11. Epub 2009/03/25. doi: 10.1037/a0015235. PubMed PMID: 19309180; PubMed Central PMCID: PMCPMCID:NA.

110. Stulz N, Lutz W, Kopta SM, Minami T, Saunders SM. Dose-effect relationship in routine outpatient psychotherapy: does treatment duration matter? Journal of counseling psychology. 2013;60(4):593-600. Epub 2013/07/03. doi: 10.1037/a0033589. PubMed PMID: 23815633; PubMed Central PMCID: PMCPMCID:NA.

111. Owen JJ, Adelson J, Budge S, Kopta SM, Reese RJ. Good-enough level and dose-effect models: Variation among outcomes and therapists. Psychotherapy Research. 2016;26(1):22-30. doi: 10.1080/10503307.2014.966346. PubMed PMID: 25346046; PubMed Central PMCID: PMCPMCID:NA.

112. Falkenstrom F, Josefsson A, Berggren T, Holmqvist R. How much therapy is enough? Comparing dose-effect and good-enough models in two different settings. Psychotherapy (Chic). 2016;53(1):130-9. Epub 2016/03/02. doi: 10.1037/pst0000039. PubMed PMID: 26928273; PubMed Central PMCID: PMCPMCID:NA.

113. Sas Institute I. SAS/STATA 14.1 Users Guide: SAS Institute, Inc; 2015.

114. Berglund P, Heeringa SG. Multiple imputation of missing data using SAS. Cary, NC: SAS Institute, Inc; 2014.

115. van Buuren S. Multiple imputation of discrete and continuous data by fully conditional specification. Statistical methods in medical research. 2007;16(3):219-42. Epub 2007/07/11. doi: 10.1177/0962280206074463. PubMed PMID: 17621469; PubMed Central PMCID: PMCPMCID:NA.

116. van Buuren S. Flexible Imputation of Missing Data. Boca Raton, FL: Chapman and Hall/CRC 2012.

117. Azur MJ, Stuart EA, Frangakis C, Leaf PJ. Multiple imputation by chained equations: what is it and how does it work? International journal of methods in psychiatric research. 2011;20(1):40-9. Epub 2011/04/19. doi: 10.1002/mpr.329. PubMed PMID: 21499542; PubMed Central PMCID: PMCPMC3074241.

118. Ibrahim JG. Short course in missing data methods in regression. 2015.

119. Ibrahim JG. Short course on missing data methods in regression, Parts I and II. 2016.

120. van Buuren S, Brand JPL, Groothuis-Oudshoorn CGM, Rubin DB. Fully conditional specification in multivariate imputation. Journal of Statistical Computation and Simulation. 2006;76(12):1049-64. doi: 10.1080/10629360600810434. PubMed PMID: :NA; PubMed Central PMCID: PMCPMCID:NA.

121. Little R, Yau L. Intent-to-treat analysis for longitudinal studies with drop-outs. Biometrics. 1996;52(4):1324-33. Epub 1996/12/01. PubMed PMID: 8962456; PubMed Central PMCID: PMCPMCID:NA.

122. Daniels MJ, Hogan JW. Missing data in longitudinal studies: Strategies for Bayesian modeling and sensitivity analysis: Chapman & Hall/CRC (Taylor & Francis Group); 2008.

123. Hedeker D, Gibbons RD. Application of random-effects pattern-mixture models for missing data in longitudinal studies. Psychological Methods. 1997;2(1):64-78. PubMed PMID: :NA; PubMed Central PMCID: PMCPMCID:NA.

124. Mallinckrodt CH. Preventing and Treating Missing Data in Longitudinal Clinical Trials: A Practical Guide: Cambridge University Press; 2013.

125. Mallinckrodt CH, Lin Q, Lipkovich I, Molenberghs G. A structured approach to choosing estimands and estimators in longitudinal clinical trials. Pharmaceutical Statistics. 2012;11:456-61. PubMed PMID: 22962024; PubMed Central PMCID: PMCPMCID:NA.

126. Mallinckrodt CH, Roger J, Chuang-Stein C, Others. Missing data: Turning guidance into action. Statistics in Biopharmaceutical Research. 2013;5(4):369-82. PubMed PMID: :NA; PubMed Central PMCID: PMCPMCID:NA.
